# A Meta-Prediction of *Methylenetetrahydrofolate-Reductase* Polymorphisms and Air Pollution Increased the Risk of Ischemic Heart Diseases Worldwide

**DOI:** 10.3390/ijerph15071453

**Published:** 2018-07-10

**Authors:** Zhao-Feng Chen, Lufei Young, Chong Ho Yu, S. Pamela K. Shiao

**Affiliations:** 1 Department of Nursing, Yuanpei University of Medical Technology, Hsinchu 30015, Taiwan; boivan@mail.ypu.edu.tw; 2 College of Nursing, Augusta University, Augusta, GA 30912, USA; luyoung@augusta.edu; 3 Department of Psychology, Azusa Pacific University, Azusa, CA 91702, USA; cyu@apu.edu

**Keywords:** meta-prediction, *methylenetetrahydrofolate reductase* gene (*MTHFR*), ischemic heart disease, air pollution

## Abstract

Ischemic heart disease (IHD) is among the leading causes of death worldwide. *Methylenetetrahydrofolate reductase (MTHFR)* polymorphisms have been associated with IHD risk, but the findings presented with heterogeneity. The purpose of the present meta-analysis was to provide an updated evaluation by integrating machine-learning based analytics to examine the potential source of heterogeneity on the associations between *MTHFR* polymorphisms and the risk of various subtypes of IHD, as well as the possible impact of air pollution on *MTHFR* polymorphisms and IHD risks. A comprehensive search of various databases was conducted to locate 123 studies (29,697 cases and 31,028 controls) for *MTHFR* C677T, and 18 studies (7158 cases and 5482 controls) for *MTHFR* A1298C. Overall, *MTHFR* 677 polymorphisms were risks for IHD (TT: Risk ratio (RR) = 1.23, *p* < 0.0001; CT: RR = 1.04, *p* = 0.0028, and TT plus CT: RR = 1.09, *p* < 0.0001). In contrast, *MTHFR* 677 CC wildtype was protective against IHD (RR = 0.91, *p* < 0.00001) for overall populations. Three countries with elevated IHD risks from *MTHFR* C677T polymorphism with RR >2 included India, Turkey, and Tunisia. Meta-predictive analysis revealed that increased air pollution was associated with increased *MTHFR* 677 TT and CT polymorphisms in both the case and control group (*p* < 0.05), with the trend of increased IHD risk resulting from increased air pollution. These results associate the potential inflammatory pathway with air pollution and the folate pathway with *MTHFR* polymorphism. Future intervention studies can be designed to mitigate MTHFR enzyme deficiencies resulting from gene polymorphisms to prevent IHDs for at-risk populations.

## 1. Introduction

Ischemic heart disease (IHD) remains the leading cause of death and disability around the world and in the United States (U.S.), accounting for 45.1% of deaths in the U.S. [1,2,3,4,5]. The estimated cost of IHD in 2012 to 2013 was $199.6 billion [4]. By the year, 2030, the medical costs of IHD are projected to increase by about 100% [4]. Coronary artery disease (CAD), a common cardiovascular disease (CVD), is also known as IHD, which includes a group of diseases across the spectrum of chronic to acute stages, including angina, acute myocardial infarction (MI), and sudden cardiac death [6]. Thus, the underlying mechanism of developing IHDs involves atherosclerosis of the blood vessels [3]. Among various risk factors, elevated plasma homocysteine level (hyperhomocysteinemia) is an independent predictor for IHD and subsequent death [7,8]. Homocysteine is positively associated with accelerating atherosclerosis, deterioration of endothelial progenitor cells, deregulating lipid metabolism, altering platelet activity, stimulating vascular smooth-muscle-cell proliferation, and inducing thrombosis [9,10,11]. The most investigated genetic variations affecting the homocysteine levels are the *methylenetetrahydrofolate reductase* (*MTHFR*) polymorphisms, which have two common variations: C677T (rs 1801133) and A1298C (rs 1801131) [12]. These two polymorphism-mutations have been associated with impaired or inactivated MTHFR enzyme and aberrant folate metabolism, leading to increased homocysteine and DNA hypomethylation [12]. Multiple meta-analysis studies also showed that *MTHFR* polymorphisms had a large effect on hyperhomocysteinemia in geographic regions of the world with low folate intakes [13,14,15].

Additional studies have supported the impact of air pollutants on the development of IHDs [16,17,18,19]. For instance, pollutants could cause the rupture of existing atherosclerotic plaque in the coronary vessels, triggering acute MI and stroke [20]. Further studies presented increased IHD-caused mortality and hospitalizations in areas with high concentrations of pollutants [17,20]. Fine particulate matters smaller than 2.5 microns (PM_2.5_) in air pollution can be inhaled into the lungs and pass through the lungs to reach cardio-pulmonary blood vessels to trigger inflammations [21]. These pollutants are mostly generated from traffic-related fuel combustion [18,20,22]. Over time, pollutants advance atherosclerosis [16,19] and consequent IHDs, which may lead to acute CAD and MI events.

Several meta-analysis studies have presented a significant association between *MTHFR* polymorphisms and IHD risk [13,14,15], while others failed to identify the association [23,24,25,26]. The discordance might be a result of the limited number of studies [23,24] or limited study populations from certain geographical areas [26,27]. Therefore, we performed an updated meta-analysis, with the addition of air pollution as a contributing factor to gene polymorphism and disease risk, by using meta-prediction analytic techniques to approach the heterogeneity of the findings and to fill the gap in the existing knowledge. In summary, we conducted a meta-prediction study using machine-learning analytics to examine (1) the associations for various IHD disease subtypes in adult populations, and (2) the impact of air pollution on the *MTHFR* polymorphisms and associated IHD risks.

## 2. Materials and Methods

### 2.1. Study Selection and Data Extraction

We conducted a comprehensive literature search from various databases, including MEDLINE through PubMed, Cochrane Library, Embase, EBSCO, Airiti Library, and WangFang databases for studies published between 1996 and 2017. This study was conducted based on the guidelines for Preferred Reporting Items of Systematic Reviews and Meta-Analysis (PRISMA) [28] and Systematic Reviews of Genetic Association Studies [29]. The search keywords and subject terms included “*methylenetetrahydrofolate reductase*” or “*MTHFR*” or “*methylenetetrahydrofolate reductase* gene polymorphisms” or “*MTHFR* polymorphisms” or “*MTHFR* variant” and “ischemic heart disease” or “IHD” or “heart disease” or “HD” or “coronary artery disease” or “CAD” or “myocardial infarction” or “MI” and “case control” or “case-control” or “meta-analysis”. We also performed cross-reference checks from previous meta-analyses (see meta-analysis reference list following Supplementary Materials Table S1). All databases were searched repeatedly at 3 different times at least 3 months apart until no additional eligible papers were identified. Two raters conducted the data collection and data extraction. The discrepancy between raters was reduced to zero prior to the data analyses.

An article was eligible if it: (1) presented the association of the *MTHFR* C677T and A1298C polymorphisms and IHD risk, with three genotype-allele counts in both the case and control groups; (2) was written in English, or (3) was written in non-English, but provided tables with genotype allele counts for both the case and control groups. IHD types, as presented in the literature for this meta-analysis, included CAD for chronic IHD and MI for acute stage IHD. The article was excluded if it: (1) did not have complete genotype allele counts in both the case and control groups, or (2) was written in non-English without tables listing the genotype allele counts.

**Figure 1:**
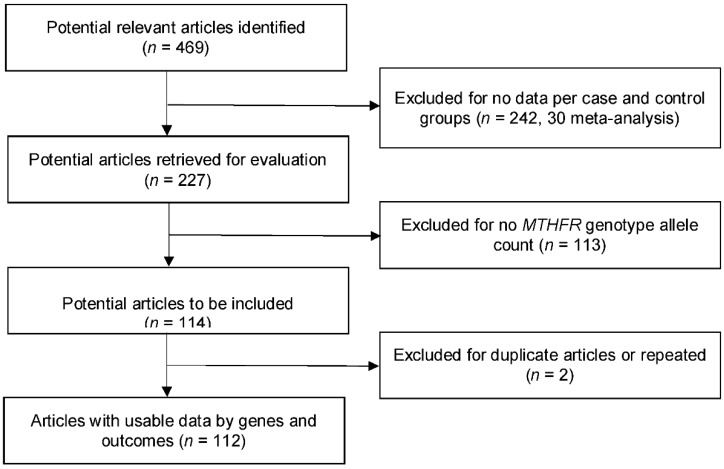
Progression of the selection of studies for the meta-analysis.

Based on the inclusion/exclusion criteria, we first identified 469 articles. A total of 242 articles were excluded because they lacked the data for the genotypes per case and control groups. Another 113 articles were eliminated due to missing or incomplete genotype allele counts. We also removed two articles because each of them reused datasets that were in another included article [30,31]. As a result, we included 112 articles, with 123 studies in the final analysis (Figure 1). Eleven additional studies are described in the following section. Four articles (Supplementary Materials reference list, 37, 51, 69, and 79, following Supplementary Materials Table S1) included both gender groups of male and female, yielding 4 additional study groups; an article (Supplementary Materials reference list, 78) included 2 IHD types (CAD and MI), yielding 1 additional study group; an article (Supplementary Materials reference list, 89) included racial-ethnic groups of African and Caucasian, and gender groups of male and female, yielding 3 additional study groups; an article (Supplementary Materials reference list, 91) with MI included two diabetes mellitus (DM) groups (with DM and non-DM), yielding 1 additional study group; an article (Supplementary Materials reference list, 93) with MI included gender groups of male and female, yielding 1 additional study group; and an article (Supplementary Materials reference list, 130) with CAD included gender groups of male and female, yielding 1 additional study group.

### 2.2. Quality Assessment

The quality of each selected study was evaluated using a quality-assessment tool based on criteria appropriate for meta-analyses [28,32]. The examined domains included external validity (10 items, score range of 0–11), internal validity (12 items, score range of 0–12), and quality of reporting (6 items, score range from 0–6). The total scores ranged from 0 to 29, with a higher score indicating higher quality [32]. The quality score of all included studies ranged from 15 to 28. Therefore, all studies reached a minimal score of 15, above the midpoint of the total possible score for the quality assessment (Supplementary Materials Table S1).

### 2.3. Data Synthesis and Analysis

The pooled risk ratios (RR), odds ratio (OR), and 95% CI for the associations of *MTHFR* polymorphisms with IHD risk were calculated. The comparison between pooled RRs and ORs indicated that RRs were more conservative, with a lower type I error [32]. Additionally, RR, as a standardized ratio, is needed for the multi-dimensional gene-environment interaction analysis in this study [32]. Therefore, RRs were used to demonstrate the associations between *MTHFR* polymorphisms and IHD risk in this report. The StatsDirect, version 3.0.193 (StatsDirect Ltd., Cambridge, UK), was used to perform pooled analyses. All *p* values were two-tailed, with a significance level at 0.05. The sensitivity analyses were performed for studies that presented significant results on the Hardy-Weinberg Equilibrium (HWE) in the genotype distribution (Supplementary Materials Table S1). For those studies that did not meet the HWE (Supplementary Materials reference list, 47 (Czech), 58 (Netherlands), 77 (U.S.), 86 (Costa Rica), 112 (China), and 128 (India)), we performed sensitivity analyses for the HWE subgroups, and found similar trends and the same directions for the risk effects. We further examined the sensitivity of including data per source of control for the healthy control versus other hospital patients, and for the quality score. Studies that met the HWE compared to those that did not, studies with a low quality score compared to those with a high score, and studies with healthy controls compared to those with other hospital patients had presented the same direction with similar risk effects. Therefore, all studies were included in the final meta-analysis, as the intent of a meta-analysis is to provide summative evidence with pooled analyses, which is the recommended approach [32,33]. An Egger’s test and funnel plots were used to detect publication bias [34,35]. A random-effects model was used in the tests of association when the heterogeneity tests were significant, with *p* < 0.05. To identify the sources of heterogeneity in the associations between *MTHFR* polymorphisms and IHD risk, we conducted subgroup analyses by geographic regions, ethnic groups, gender, IHD sub-types, air pollution as an environmental factor, sources of control, and quality score. We used the JMP 13 Pro program (SAS Institute, Cary, NC, USA) to examine the gene-air pollution interaction on IHD risk [36,37], and generated the geographic information system (GIS) maps to visualize the geographic distribution and pattern of *MTHFR* polymorphisms and their associations with IHD risks [38]. These GIS maps were drawn by country distribution (through the graph builder in the JMP Pro program per outcomes of interest, such as percent polymorphism or IHD risks) and were helpful for visually identifying geographic patterns, and to manage the geospatial data.

We applied recursive partition trees in the JMP Pro 13 program to examine how an independent variable (e.g., air pollution) can make a decisive split of the data by partitioning the groups (such as air pollution levels) into the pairs of subgroups with reference to the dependent variable (the percentage of polymorphisms and IHD risks). The recursive partition tree does this by exhaustively searching all possible groupings [36]. These partitions of the data are done recursively, forming a tree of decision rules until the desired fit is reached. The partition process is driven by the Gini impurity criterion, which is based upon information theory [39,40]. Gini is a measure of group impurity, which is the inverse of group homogeneity, and, thus, a smaller Gini is better. The goodness of the partition result can be judged by using the Akaike’s information criterion correction (AICc). A smaller AICc suggests less complexity and a better model for optimization (a balance between fitness and parsimony) [41,42,43]. We entered the air-quality data for various countries using the guidelines from the World Health Organization on air quality measures, the death rates from air pollution (AP death) (Level 1: <50 deaths per million, Level 2: 51–100 deaths per million, Level 3: 101–250 deaths per million, Level 4: 251–400 deaths per million, and Level 5: >401 deaths per million of population) [44,45]. We further verified these levels with current scales on air pollution data [46,47,48,49], and used the most complete and current scaled air pollution data for the analyses. Only one study from Ireland (Supplementary Materials reference list, 72) presented a Level 1 air pollution level, thus, we combined Level 1 and Level 2 together for the grouping analysis.

Both GIS maps and recursive partition trees are common machine-learning analytical techniques for handling multidimensional and/or large-scale datasets. Different from conventional hypothesis testing, machine-learning or big-data analytics does not start with a pre-determined hypothesis. Rather, data-driven pattern recognition plays the central role. To triangulate and cross-validate the findings, we used machine-learning based validation analytics, including partition trees, and nonlinear-association fit modeling to explore the sources of heterogeneity in addition to the conventional approaches [32,36]. We used a bi-variate nonlinear fit plot to visualize the distribution patterns based on air pollution levels across countries on the pooled percentages of *MTHFR* genotypes per case and control groups, and IHD risks to cross-validate the results [32]. To compare AICc results with the partition trees, we used the Tukey’s test [36] to examine whether partition trees and Tukey tests concurred with each other. The aim of the meta-predictive analysis was to generate more precise predictions by integrating data from multidimensional sources. The main purpose for using both conventional statistical and machine learning (e.g., recursive partition trees) methods is to verify the results by cross-validation, including AICc, yielding a more accurate meta-prediction.

## 3. Results

### 3.1. Sample Characteristics and Genotype Frequency

The most investigated racial or ethnic populations in these studies were Caucasian (60 studies), followed by East Asian (32 studies), South Asian (13 studies), Middle-Eastern (7 studies), African (6 studies), mixed groups (3 studies), and Hispanic (2 studies). Among the 123 case-control studies, 93 studies were conducted in CAD patients and 30 in MI patients (Table 1 and Table 2). No significant association was found between *MTHFR* 1298 polymorphism and IHD risk. The frequencies of the *MTHFR* 677 homozygous TT genotype were highest in Hispanic populations (28.90%), followed by East Asian (20.23%), African (12.01%), Caucasian (11.10%), Middle Eastern (10.28%), mixed group (9.78%), and South Asian (3.76%). For pooled analyses in *MTHFR* 1298, the frequencies of the homozygous CC genotype were highest in the mixed group (27.36%), South Asian (16.55%), Caucasian (10.40%), African (6.86%), Middle Eastern (6.4%), and East Asian (3.70%) populations (Supplementary Materials Figure S1a). The frequencies of *MTHFR* 1298 polymorphisms are presented in the Supplementary Materials Figure S1b.

### 3.2. Pooled Meta-Analysis

Table 1 presents the schema of the significant findings across IHD types with CAD and MI subtypes for the *MTHFR* 677 genotypes and risk of IHD; Table 2 presents the results of the detailed analysis. For analyses of the IHD subtypes of CAD and MI, detailed results are presented in the Supplementary Materials Tables S2 and S3. For all included study groups, the pooled analysis presented *MTHFR* 677 polymorphisms as risks for IHD (TT: Risk ratio (RR) = 1.23, *p* < 0.0001; CT: RR = 1.04, *p* = 0.0028, and TT plus CT: RR = 1.09, *p* < 0.0001), with the T allele having an 11% greater risk of developing IHD (RR = 1.11, *p* < 0.0001). In contrast, *MTHFR* 677 CC wildtype (RR = 0.91, *p* < 00001) was protective against IHD, and holders of the C allele would have a 4% lesser chance of developing IHD (RR = 0.96, *p* <0.0001) (Table 2). The results of the pooled odds ratio (OR) for the *MTHFR* 677 polymorphisms presented greater ratios, as compared to the RRs, for IHD risk (TT: OR = 1.28, *p* < 0.0001; CT: OR = 1.08, *p* = 0.0012, and TT plus CT: OR = 1.22, *p* < 0.0001), with the T allele having a 17% greater odds of developing IHD (OR = 1.17, *p* < 0.0001) versus the CC wildtype (OR = 0.82, *p* < 00001), which presented a lesser odds as compared to RR against IHD, with holders of the C allele having a 15% lesser odds of developing IHD (OR = 0.85, *p* < 0.0001). Hence, the comparison between the pooled RRs and ORs indicated that RRs were more conservative, with a lower type I error [32]. As the RR is used for the standardized ratio in the multi-dimensional gene-environment interaction analysis [32], we used RRs to demonstrate the associations between *MTHFR* polymorphisms and IHD risk in this report.

### 3.3. Subgroup Analysis by Ethnicity and Countries

**Figure 2:**
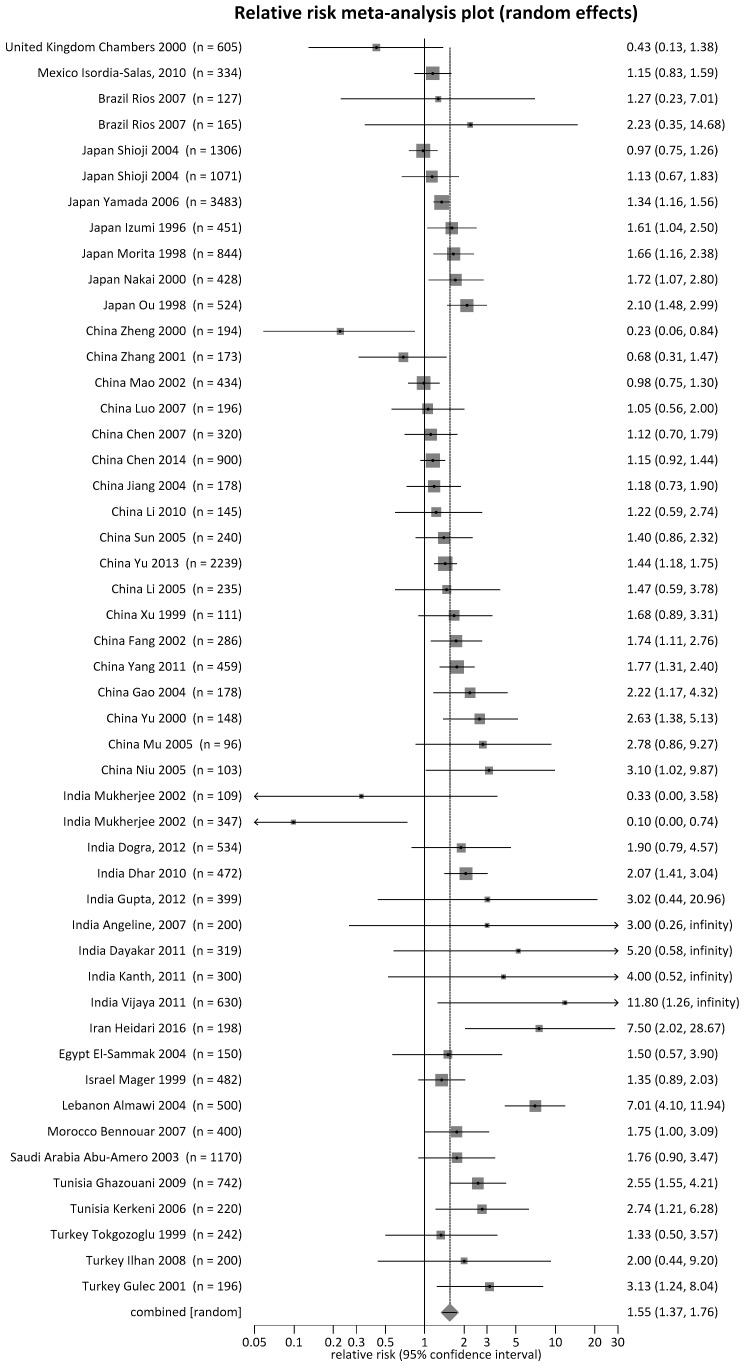
Forest plot for the meta-analysis of *MTHFR* 677 polymorphism by TT genotype, countries of non-Caucasian ethnicity with risks >1.

To identify sources of heterogeneity, subgroup analysis per ethnic groups showed that *MTHFR* 677 TT and CT genotypes were associated with IHD risk. The ranking of IHD risk with the *MTHFR* 677 TT genotype from high to low according to ethnic groups was Middle Eastern (RR = 2.62), African (RR = 2.14), South Asian (RR = 1.51), and East Asian (RR = 1.31); and with the CT genotype, the ranking of risks was South Asian (RR = 1.32) and Middle Eastern (RR = 1.18) (all *p* < 0.01). It is worthy to point out that the Middle Eastern and African samples had RR > 2, and, as in biological studies, RR > 2 could infer causality [33,50,51,52]. On the other hand, the *MTHFR* 677 CC wildtype played a protective role against IHD and ranked as follows: Middle Eastern (RR = 0.76), East Asian (RR = 0.84), and South Asian (RR = 0.91) (all *p* < 0.01) (Table 2). For the subgroup analysis by countries, we further examined whether *MTHFR* 677 TT genotypes posed a risk (RR > 1), were protective (RR < 1), or had a mixed effect (RR ~ 1) (Table 2). The countries that had *MTHFR* 677 TT as a risk genotype included Australia, European countries (Poland, Russia, Slovakia, Sweden, Croatia, Czech Republic, Netherlands, Switzerland, France, Portugal, United Kingdom, and Ireland), Canada, South America (Brazil and Mexico), Asian countries (Japan, China, and India), Middle-Eastern countries (Israel, Turkey, Iran, Egypt, Saudi Arabia, and Lebanon), and African countries (Tunisia and Morocco) (Supplementary Materials Figure S2a). It is worthy to point out the countries with RR > 2 because, as in biological studies, RR > 2 could infer causality [33,50,51,52]. These countries with elevated risks included India, Turkey, and Tunisia (Figure 2, forest plots). In Figure 2, six studies from India presented a very wide range of the 95% confidence interval (CI) (Supplementary Materials reference list, 125–127, 129, and 130, with both gender groups of male and female), with four studies presenting zero counts on the *MTHFR* 677 TT genotype in their control groups, and two studies (Supplementary Materials reference, 130, included two gender groups of male and female) presenting zero counts on the *MTHFR* TT genotype in the case groups. The countries that presented *MTHFR* 677 TT as a protective genotype (RR < 1) included three European countries (Hungary, Italy, and Spain), one Central American country (Costa Rica), two Asian countries (South Korea and Pakistan), and South Africa (study population consisted of descendants of Indian immigrants) (Supplementary Materials Figure S2b). For Germany, United States, and Taiwan, the *MTHFR* 677 TT genotype presented a mixed IHD risk (Supplementary Materials Figure S2c).

### 3.4. Subgroup Analysis by IHD Subtypes

We also analyzed subgroups by the IHD subtypes of CAD and MI to present the source of heterogeneity. For CAD subtype, risk genotypes were *MTHFR* 677 TT (RR = 1.27, *p* < 0.0001), CT (RR = 1.04, *p* = 0.0074), and TT plus CT (RR = 1.09, *p* < 0.0001). In contrast, the *MTHFR* 677 CC genotype played a protective role against CAD (RR = 0.91, *p* < 0.0001). Likewise, the *MTHFR* 677 T allele increased the risk of CAD by 12% (RR = 1.12, *p* < 0.0001), while its C allele could reduce CAD risk by 5% (RR = 0.95, *p* < 0.0001). Further subgroup analyses per ethnic subgroups revealed that the *MTHFR* 677 TT and CT genotypes were associated with CAD risk in Caucasian, East Asian, South Asian, Middle-Eastern, and African groups, with RRs ranging from 1.11 to 2.56 for TT, and 1.18 to 1.36 for CT. On the other hand, the *MTHFR* 677 CC wildtype played a protective role against CAD in East Asian, South Asian, and Middle-Eastern groups, with RRs ranging from 0.76 to 0.89 (Supplementary Materials Table S2). For the MI subtype, *MTHFR* 677 TT plus CT were risk genotypes (RR = 1.06, *p* = 0.0187), while the CC genotype played a protective role against MI (RR = 0.93, *p* = 0.0148). Individuals with the *MTHFR* 677 T allele had a 6% higher risk of having MI (RR = 1.06, *p* < 0.0073), while those with the *C* allele had a 3% lower chance of developing MI (RR = 0.97, *p* = 0.0223). Further subgroup analyses by ethnic groups showed that East Asian groups with the TT genotype had an increased risk of developing MI (RR = 1.30, *p* < 0.0001) (Supplementary Materials Table S3).

### 3.5. Heterogeneous Findings by GIS Map

To validate the heterogeneous findings, we further utilized a geographic information system to visualize the variability in regional distributions [53]. The global maps presented variations in the distribution of *MTHFR* 677 polymorphism rates and their roles in IHD risk across regions (Supplementary Materials Figure S3a,b). In the first two GIS maps, the continuous color spectrum from yellow to red represented the increasing rates of TT and TT plus CT genotypes, and, in the third map, the red-to-green color spectrum demonstrated a risk gradient, with red indicating IHD risk and green indicating protective effects. Consistent with the aforementioned subgroup analyses, GIS maps presented that *MTHFR* 677 TT and TT plus CT increased the risk of IHD in most countries except Italy, Spain, and South Korea.

### 3.6. Meta-Prediction: MTHFR Polymorphisms and Air Pollution Associated with Risk of IHD

**Figure 3:**
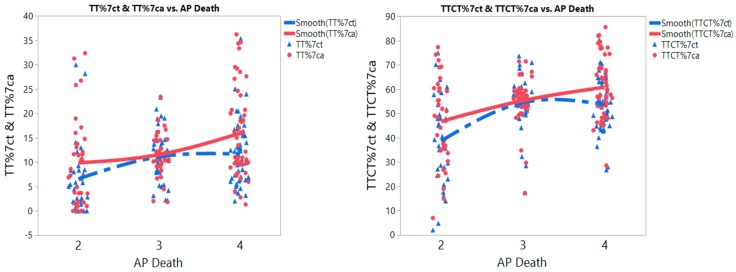
Nonlinear curve fits of *MTHFR* 677 polymorphism TT (**left**) and TT plus CT (**right**) genotypes of control and ischemic heart disease (IHD) groups with annual death rates from air pollution (AP death) per million population (levels: 2 = 51–100 deaths/million, 3 = 101–250 deaths, 4 = 251–400 + deaths); case = red, control = blue. Note. TT%7ct: Percentage of *MTHFR* 677 TT genotype in control group; TT%7ca: Percentage of TT genotype in IHD group; TTCT%7ct: Percentage of TT plus CT genotypes in control group; TTCT%7ca: Percentage of TT plus CT genotypes in IHD group.

For meta-prediction, we performed both a partition tree analysis and a Tukey’s test to examine the potential interaction between independent variables, such as levels of air pollution as measured by the death rates associated with air pollution per country (AP), source of controls, and the quality score, with outcome variables of *MTHFR* polymorphisms and IHD risk. We did not find significance with other factors except for the AP on the interaction between polymorphisms and IHD risks. The annual AP by country was reported and classified by the World Health Organization (WHO) using the following criteria: (1) Level 2 = 51–100 deaths/million population (DMP); (2) Level 3 = 101–250 DMP; and (3) Level 4 = 251–400 or greater DMP [49]. We present the partition tree (split groups) and Tukey’s test results side by side for the *MTHFR* 677 genotypes and AP (Table 3). The meta-prediction using the partition tree and the Tukey’s tests were not performed for the *MTHFR* 1298 genotypes because that genotype was rarely studied to date. There were significant increases in the percentages of *MTHFR* 677 TT and CT polymorphism between AP levels 2 and 3 (*p* < 0.05), and levels 2 and 4 (*p* < 0.01) for both the case and control groups. The partition tree results, depicted in Table 3, indicate a clear and consistent pattern: No matter what the variable is (TT%ct, TT%ca, CT%ct, CT%ca, etc.), the mean percent of polymorphisms of TT or CT were lower with lower levels of AP death, and increased with higher levels of AP death. In addition, strikingly higher polymorphisms were noted as AP rates increased for the IHD case group as compared to the corresponding control group. In contrast, the percentages of the *MTHFR* 677 CC wildtype decreased with increased levels of AP death for both the case and control groups. The risks of IHD from *MTHFR* 677 TT plus CT polymorphisms presented a greater risk with lower levels of AP death, and was significantly higher between AP levels 2 and 3 (*p* < 0.001), and levels 2 and 4 (*p* < 0.05). It is important to point out that, unlike ANOVA, in which levels are grouped by the analyst, the partition done by the decision tree is data-driven i.e., the algorithm determines what partition could result in better purity (homogeneity), meaning that the associational pattern is less likely to be a statistical artifact capitalized on chance. The nonlinear curves were further revealing of the differences on the percentage of *MTHFR* polymorphisms at different AP levels (Figure 3). With a change in AP levels from low (Level 2) to high (Level 3 and 4).

There was a substantial increase in the percentages of *MTHFR* 677 polymorphisms for both TT and TT plus CT genotypes in both case and control groups.

## 4. Discussion

In summary, we have updated and performed the pooled analyses with 123 studies of 29,697 IHD cases and 31,028 controls for *MTHFR* 677, and 18 studies of 7158 IHD cases and 5482 controls for *MTHFR* 1298 polymorphisms, adding the environmental air pollution factor. We extended the findings of previous meta-analyses [26,54,55,56], with a significant association between *MTHFR* 677 polymorphism and the risk of developing IHD identified. A great deal of heterogeneity was noted across geographic areas and populations based on the IHD subtypes of CAD and MI. To expand the findings from previous meta-analyses, we further conducted a meta-prediction to examine a potential interaction between air pollution and *MTHFR* polymorphisms on IHD risk. The nonlinear curve plots demonstrated that the percentages of *MTHFR* 677 polymorphism in both the case and control groups increased substantially in regions with higher levels of air pollution. Compared to control groups, the case groups showed higher percentages of *MTHFR* 677 TT and CT polymorphisms where the level of air pollution was higher. These findings are consistent with prior studies that found significantly increased *MTHFR* gene polymorphisms with increased air pollution levels in various diseases, including various cancers, Alzeimer’s disease, and hypertensive disorders during pregnancy (HDP) [32,57,58,59,60]. Also, an increased air pollution level was associated with *MTHFR* polymorphism associated with HDP risk [57].

To examine possible sources of heterogeneity, we examined data using other factors that became available with this study, including air pollution, HWE status (met or unmet), quality score (high or low), and the sources of controls (healthy control or inpatients as controls). We observed similar trends of these factors on the polymorphism and IHD risks, with the same directions for the risk effects. For the meta-prediction analysis, we examined the effects of air pollution, sources of controls, and quality score status on the polymorphism and IHD risks. Continuing the findings from previous studies [32,57,58,59,60], air pollution, as measured by the AP, was the only significant factor for the polymorphism and disease risks. It is worth noting the limitations of the differences found in Table 3 that may be caused by additional factors, such as cultural, economic, or industrial factors, but not from air pollution. In addition, despite the potential risk of ecological fallacy, in some cases, global data must be used because collecting individual data is impossible. Air pollution is a typical example. Although techniques of monitoring how much pollutant is absorbed by each individual has been under development by taking the immediate surroundings, individuals’ biophysical characteristics, and individuals’ space-time activities, such as culture, economic, or industrial factors, into account [61], these methods are not prevalent across countries. 

Previous studies have shown that air pollution directly harms the cardiovascular system [18,62]. The association of environmental pollution with increased polymorphism rates is alarming for human health. The potential detriments of this association could be exacerbated by the greenhouse effect from air pollution because heat can further diminish the functioning of MTHFR enzymes. Decreased MTHFR enzyme functioning can compromise methylation pathways and result in elevation of plasma homocysteine, an independent risk factor for IHD [63,64]. Furthermore, both *MTHFR* 677 polymorphisms and air pollution may compound the detriments of hyperhomocysteinemia to compromise the epigenetic health status for persons with IHD. 

For the meta-predictive analysis, meta-regression using linear modeling, such as Pearson’s correlation and ordinary least squares regression, has been criticized as overly simplistic. When the underlying data structure is nonlinear, such as the association between death from air pollution with polymorphism and IHD risk, then nonlinear modeling is more appropriate [64,65,66]. Therefore, we further demonstrated the association of *MTHFR* 677 polymorphism with IHD risks using machine learning meta-predictive analytics, including GIS maps, to present the heterogeneity of polymorphisms worldwide. The great heterogeneity in the relationship between *MTHFR* 677 polymorphism and IHD risk across ethnic groups and regions may be attributed to (1) the human migration paths and variations in the percentages of *MTHFR* 677 polymorphism among different ethnic groups across countries; (2) various IHD disease subtypes; (3) various levels of air pollution across different geographic areas; and (4) differences in lifestyles (e.g., eating behaviors and food choices). For instance, in countries where people are acculturated to eat plenty of vegetables, diet may have modified the effects of *MTHFR* 677 polymorphisms. Korea has a well-known taste for fermented cabbage, and Mediterranean cuisines, such as Italian or Spanish, include plants, such as peppers, tomatoes, eggplant, and zucchini; in such cultures, *MTHFR* 677 polymorphism was surprisingly protective against IHD [67,68]. *MTHFR* 677 polymorphism had a significant association with CAD, but not acute MI, in this analysis. This lack of significance for MI conditions could be due to a smaller number of studies (30 studies); perhaps because it might also be more feasible to conduct studies during the chronic disease state, such as CAD (93 studies), than during the acute MI stage. An additional potential explanation has been posited that homocysteine is considered an endothelial toxin that promotes thrombotic tendency and atherosclerosis for the pathogenesis of CAD, but not for that of acute MI [13]. To solidify the effects of *MTHFR* 677 polymorphism risk across populations, additional studies with purposeful designs and interventions can be helpful.

Despite the clear strengths of our study, some additional limitations merit some consideration. First, the data for potential confounding factors (e.g., cultural, economic, or industrial factors) were not provided in original studies, therefore, further investigations are needed to determine whether the differences in the magnitude of association between air pollution with *MTHFR* polymorphism and IHD risk are attributed to air pollution or other confounding factors. Second, to provide comprehensive pooled analyses, we included studies with small sample sizes and a wide range of 95% CI, with some studies that did not meet the HWE. However, based on our sensitivity analysis by the HWE status (met vs. unmet), including underpowered studies had no effect on the direction of effects based on *MTHFR* polymorphisms and IHD risks. Therefore, we included all studies in the meta-analyses, as the intent of meta-analysis is to provide summative filtered evidence with pooled analyses. Given the limitation and quality of original studies, future studies are needed to continue examining the effects of gene-environment interactions from air pollution and gene polymorphisms on health outcomes across the disease spectrum. The results of heightened disease risks from air pollution and gene polymorphisms for IHD could bring attention to the development of health policy for clean air environment and interventions to mitigate enzyme deficiency in folate metabolism pathways to prevent IHD.

## 5. Conclusions

*MTHFR* polymorphisms were risks for IHD and for gene-environment interactions, with the 677 wild type plying a greater protective role in countries with higher air pollution levels than in those countries with lower air pollution levels. Epigenetic factors, including environmental toxicants from air pollution and healthy lifestyles, such as food choices to detox, may affect the development of IHDs by modifying gene expressions in methylation pathways. Proactive strategies could be implemented in geographic regions with significant air pollution levels to mitigate the effects of MTHFR enzyme deficiencies that are a result of polymorphisms to prevent IHDs and to promote the health of susceptible, at-risk populations.

## Figures and Tables

**Table 1:** Schema of significant findings across studies on *MTHFR* 677 genotypes and risk of ischemic heart disease (IHD).

*MTHFR* 677	IHD	CAD	MI
	(*n* Case/*n* Control)	(*n* Case/*n* Control)	(*n* Case/*n* Control)
	123 Studies (29,697/31,028)	93 Studies (22,994/20,221)	30 Studies (6709/10,807)
**Overall**	Risk Type: TT, CT	Risk Type: TT, CT	
	Protective: CC	Protective: CC	Protective: CC
**Subgroups**			
**Caucasian**	60 Studies	47 Studies	13 Studies
	(22,042/14,925)	(13,817/10,702)	(2850/4223)
	Risk trend: TT	Risk Type: TT	NS
	Protective trend: CC	Protective: CC	
**East Asian**	32 Studies	25 Studies	7 Studies
	(7112/9471)	(4855/4931)	(2257/4540)
	Risk Type: TT	Risk Type: TT	Risk Type: TT
	Protective: CC	Protective: CC	
**South Asian**	13 Studies	8 Studies	5 Studies
	(2475/2763)	(1447/1621)	(1028/1142)
	Risk Type: TT, CT	Risk Type: TT, CT	NS
	Protective: CC	Protective: CC	
**Mixed group**	3 Studies	2 Studies	1 Study
	(951/852)	(882/514)	(69/338)
	NS	NS	--
**Middle Eastern**	7 Studies	6 Studies	1 Study
	(1265/1723)	(1169/1623)	(99/100)
	Risk Type: TT, CT	Risk Type: TT, CT	--
	Protective: CC	Protective: CC	
**Hispanic**	2 Studies	--	2 Studies
	(353/364)		(353/364)
	NS		NS
**African**	6 Studies	5 Studies	1 Study
	(874/930)	(824/648)	(50/282)
	Risk Type: TT	Risk Type: TT, CT	--

Note. NS: No statistical significance; --: Not available or not applicable; CAD: Coronary artery disease, MI: Myocardial infarction.

**Table 2:** Pooled analyses of *MTHFR* 677 genotypes and risks of ischemic heart disease (IHD) by ethnicity (123 Studies).

Genotype per Ethnicity (n Studies)	IHD Cases (*n* = 29,697) *n* (%)		Controls (*n* = 31,028) *n* (%)		Test of Heterogeneity			Test of Association	
					Q	*p* *	*I* ^2^	Risk Ratio (95% CI)	*p*
**TT (123)**	3812	(12.84)	3330	(10.73)	238.56	<0.0001	48.9%	1.23 [1.14, 1.33]	<0.0001
Caucasian (60)	1850	(11.10)	1479	(9.91)	59.38	0.4615	0.6%	1.07 [1.00, 1.14]	0.0528
East Asian (32)	1439	(20.23)	1443	(15.24)	60.75	0.0011	49%	1.31 [1.17, 1.47]	<0.0001
South Asian (13)	93	(3.76)	71	(2.57)	20.19	0.0635	40.6%	1.51 [1.13, 2.01]	0.0048
Mixed Group (3)	93	(9.78)	88	(10.33)	0.05	0.9771	0%	0.86 [0.63, 1.17]	0.33
Middle Eastern (7)	130	(10.28)	89	(5.17)	27.38	0.0001	78.1%	2.62 [1.37, 5.01]	0.0034
Hispanic (2)	102	(28.90)	106	(29.12)	1.50	0.2204	33.4%	0.99 [0.79, 1.25]	0.94
African (6)	105	(12.01)	54	(5.81)	2.03	0.8454	0%	2.14 [1.56, 2.94]	<0.0001
**CT (123)**	12,777	(43.02)	12,815	(41.30)	174.49	0.0013	30.1%	1.04 [1.01, 1.07]	0.0028
Caucasian (60)	7402	(44.41)	6448	(43.20)	47.63	0.8555	0%	1.01 [0.98, 1.04]	0.48
East Asian (32)	3338	(46.93)	4439	(46.87)	53.81	0.0067	42.4%	1.03 [0.98, 1.09]	0.27
South Asian (13)	634	(25.62)	531	(19.22)	27.60	0.0063	56.5%	1.32 [1.12, 1.57]	0.0011
Mixed Group (3)	415	(43.64)	327	(38.38)	1.44	0.4874	0%	1.02 [0.90, 1.15]	0.78
Middle Eastern (7)	489	(38.66)	593	(34.42)	5.67	0.4607	0%	1.18 [1.07, 1.30]	0.0011
Hispanic (2)	165	(46.74)	157	(43.13)	1.88	0.1709	46.7%	1.08 [0.92, 1.27]	0.34
African (6)	334	(38.22)	320	(34.41)	9.31	0.0975	46.3%	1.13 [1.00, 1.28]	0.0547
**CC (123)**	13,108	(44.14)	14,883	(47.97)	269.85	<0.0001	54.8%	0.91 [0.89, 0.94]	<0.0001
Caucasian (60)	7415	(44.49)	6998	(46.89)	63.45	0.3225	7%	0.98 [0.95, 1.00]	0.058
East Asian (32)	2335	(32.83)	3589	(37.89)	89.99	<0.0001	65.6%	0.84 [0.77, 0.91]	<0.0001
South Asian (13)	1748	(70.63)	2161	(78.21)	44.14	<0.0001	72.8%	0.91 [0.86, 0.96]	0.0011
Mixed Group (3)	443	(46.58)	437	(51.29)	1.09	0.5804	0%	1.02 [0.92, 1.12]	0.76
Middle Eastern (7)	646	(51.07)	1041	(60.42)	16.22	0.0126	63%	0.76 [0.66, 0.87]	<0.0001
Hispanic (2)	86	(24.36)	101	(27.75)	0.04	0.8487	0%	0.88 [0.69, 1.13]	0.31
African (6)	435	(49.77)	556	(59.78)	11.88	0.0365	57.9%	0.87 [0.76, 1.00]	0.0501
**TT + CT (123)**	16,589	(55.86)	16,145	(52.03)	283.27	<0.0001	56.9%	1.09 [1.06, 1.11]	<0.0001
**CC + CT (123)**	25,885	(87.16)	27,698	(89.27)	304.35	<0.0001	59.9%	0.98 [0.97, 0.99]	<0.0001
**T (123)**	10,200	(34.35)	9737	(31.38)	180.11	0.0005	32.3%	1.11 [1.07, 1.14]	<0.0001
**C (123)**	19,496	(65.65)	21,290	(68.62)	172.23	0.0019	29.2%	0.96 [0.94, 0.97]	<0.0001
**Subgroups**									
**TT Risk > 1 (87) ^1^**	18,032	(60.72)	22,413	(72.23)					
TT	2515	(13.95)	2351	(10.49)	163.42	<0.0001	47.4%	1.40 [1.28, 1.53]	<0.0001
CT	7720	(42.81)	9243	(41.24)	148.57	<0.0001	42.1%	1.05 [1.02, 1.09]	0.0029
CC	7797	(43.24)	10,819	(48.27)	208.20	<0.0001	58.7%	0.88 [0.85, 0.91]	<0.0001
TT + CT	10,235	(56.76)	11,594	(51.73)	225.28	<0.0001	61.8%	1.13 [1.09, 1.17]	<0.0001
CC + CT	15,517	(86.05)	20,062	(89.51)	267.73	<0.0001	67.9%	0.96 [0.95, 0.98]	<0.0001
T	6375	(35.35)	6972	(31.11)	134.06	0.0007	35.8%	1.17 [1.12, 1.22]	<0.0001
C	11,657	(64.65)	15,440	(68.89)	128.35	0.0021	33%	0.94 [0.92, 0.95]	<0.0001
**TT Risk < 1 (12) ^2^**	2731	(9.20)	2156	(6.95)					
TT	322	(11.79)	299	(13.87)	7.89	0.7229	0%	0.86 [0.75, 1.00]	0.051
CT	1151	(42.15)	848	(39.33)	8.71	0.6489	0%	1.04 [0.97, 1.12]	0.23
CC	1258	(46.06)	1009	(46.80)	11.75	0.3828	6.4%	1.00 [0.95, 1.06]	0.90
TT + CT	1473	(53.94)	1147	(53.20)	11.24	0.4235	2.1%	1.00 [0.95, 1.05]	0.90
CC + CT	2409	(88.21)	1857	(86.13)	13.30	0.2745	17.3%	1.02 [1.00, 1.04]	0.052
T	897.5	(32.86)	723	(33.53)	6.66	0.8262	0%	0.97 [0.90, 1.05]	0.44
C	1833.5	(67.14)	1433	(66.47)	7.30	0.7742	0%	1.02 [0.98, 1.06]	0.44
**TT Risk~1 (24) ^3^**	8934	(30.08)	6459	(20.82)					
TT	975	(10.91)	680	(10.53)	18.02	0.7562	0%	0.99 [0.90, 1.09]	0.86
CT	3906	(43.72)	2724	(42.17)	16.19	0.8468	0%	1.01 [0.97, 1.05]	0.73
CC	4053	(45.37)	3055	(47.30)	17.43	0.7875	0%	1.00 [0.96, 1.03]	0.81
TT + CT	4881	(54.63)	3404	(52.70)	16.51	0.8325	0%	1.00 [0.97, 1.04]	0.81
CC + CT	7959	(89.09)	5779	(89.47)	18.51	0.7295	0%	1.00 [0.99, 1.01]	0.86
T	2928	(32.77)	2042	(31.61)	8.89	0.9963	0%	1.00 [0.95, 1.05]	0.95
C	6006	(67.23)	4417	(68.39)	9.19	0.9953	0%	1.00 [0.98, 1.02]	0.95

*Note.* Q = Cochran’s Q; CI = Confidence interval; *p* * for random effects model; ^1^ countries with *TT* risks >1: Australia, Poland, Russia, Slovakia, Sweden, Croatia, Czech Republic, Netherlands, Switzerland, France, Portugal, United Kingdom, Canada, Ireland, Mexico, Brazil, Japan, China, India, Iran, Egypt, Israel, Lebanon, Morocco, Saudi Arabia, Tunisia, Turkey; ^2^ countries with TT with risks <1: Hungary, Italy, Spain, Costa Rica, South Korea, Pakistan, South Africa (Indian descendants); ^3^ countries with TT risks varied around 1: Germany, United States, Taiwan.

**Table 3:** Meta-prediction: Death from air pollution (AP death) on percentages of *MTHFR* 677 genotypes for control (Ct) and ischemic heart disease (IHD) cases (Ca) and IHD risks.

Variable	Partition Tree					Tukey’s Test					
	AICc	AP Death Levels	Count	Mean	SD	Levels Compared	Difference	SE Difference	Lower CI	Upper CI	*p*
TT%ct	802	2	34	6.6	7.0	4/2	5.1	1.4	1.8	8.4	0.0011
		3 and 4	89	11	6.0	3/2	4.5	1.5	1.1	8.0	0.0064
						4/3	0.6	1.3	−2.6	3.7	0.91
TT%ca	857	2 and 3	74	11	6.8	4/2	6.1	1.7	1.9	10	0.0019
		4	49	16	9.0	4/3	4.4	1.7	0.5	9.3	0.0251
						3/2	1.7	1.8	−2.6	6.0	0.63
CT%ct	894	2	34	32	13	3/2	11	2.1	6.3	16	<0.0001
		3 and 4	89	43	7.0	4/2	9.9	2.0	5.1	15	<0.0001
						3/4	1.4	1.9	−3.2	6.0	0.75
CT%ca	887	2	34	39	12	4/2	8.0	2.0	3.3	13	0.0002
		3 and 4	89	44	6.9	3/2	6.9	2.1	2.0	12	0.0031
						4/3	1.1	1.9	−3.3	5.6	0.82
CC%ct	983	2	34	61	18	2/3	16	3.0	8.6	23	<0.0001
		3 and 4	89	46	11	2/4	15	2.9	8.1	22	<0.0001
						4/3	0.8	2.8	−5.7	7.4	0.95
CC%ca	997	2	34	53	18	2/4	14	3.0	6.9	21	<0.0001
		3 and 4	89	42	12	2/3	8.6	3.2	1.1	16	0.0211
						3/4	5.5	2.9	−1.3	12	0.14
TT + CT%ct	983	2	34	39	18	3/2	16	3.0	8.6	23	<0.0001
		3 and 4	89	54	11	4/2	15	2.9	8.1	22	<0.0001
						3/4	0.8	2.8	−5.7	7.4	0.9499
TT + CT%ca	997	2	34	47	18	4/2	14	3.0	6.9	21	<0.0001
			89	58	12	3/2	8.6	3.2	1.1	16	0.0211
						4/3	5.5	2.9	−1.3	12	0.1410
RRTT	373	2	29	1.8	1.7	2/3	0.6	0.3	−0.1	1.2	0.11
		3 and 4	89	1.4	0.9	2/4	0.3	0.3	−0.3	1.0	0.42
						4/3	0.2	0.3	−0.4	0.8	0.60
RRCT	137	2	34	1.3	0.8	2/3	0.3	0.1	0.1	0.6	0.0031
		3 and 4	89	1.1	0.2	2/4	0.3	0.1	0.03	0.5	0.0244
						4/3	0.1	0.1	−0.1	0.3	0.65
RRTT + CT	156	2	34	1.4	0.8	2/3	0.4	0.1	0.2	0.6	0.0007
		3 and 4	89	1.1	0.2	2/4	0.3	0.1	0.04	0.5	0.0200
						4/3	0.1	0.1	−0.1	0.4	0.40
RRCC	−84	2 and 3	74	0.9	0.2	3/4	0.2	0.04	0.1	0.2	0.0001
		4	49	0.9	0.2	3/2	0.1	0.04	0.03	0.2	0.0041
						2/4	0.02	0.04	−0.1	0.1	0.81

*Note.* CI = confidence interval; AICc = Akaike’s information criterion correction; AP death levels = annual death rates from air pollution levels per million (Levels 2 = 51–100, 3 = 101–250, 4 = 251–400 and greater); RR = risk ratio; ct = controls; ca = IHD case; TT%ct = percentages of *MTHFR* 677 TT genotype in control group; TT%ca = percentages of TT genotype in IHD cases; CT%ct = percentages of CT genotype in control group; CT%ca = percentages of CT genotype in IHD cases; CC%ct = percentages of CC wildtype in control group; CC%ca = percentages of CC wildtype in IHD cases; TTCT%ct = percentages of TT plus CT genotypes in control group; TTCT%ca = percentages of TT plus CT genotypes in IHD cases; RRTT = risk ratio of TT; RRCT = risk ratio of CT; RRTT + CT = risk ratio of TT plus CT; RRCC = risk ratio of CC.

## Supplementary Materials

The following are available online at http://www.mdpi.com/1660-4601/15/7/1453/s1, Table S1: Summary of participant characteristics and *MTHFR* 677 and 1298 loci distributions for studies on ischemic heart disease (IHD) included in this review, by geographic location (112 articles, 123 study groups based on counts available from both control and IHD groups). Table S2: Pooled analysis: *MTHFR* C677T genotypes and risks of coronary artery disease (CAD) by ethnicity (93 Studies). Table S3: Pooled analysis: *MTHFR* C677T genotypes and risks of myocardial infarction (MI) by ethnicity (30 Studies). Figure S1a. *MTHFR* C677T percentage of polymorphism per control and IHD case groups; Figure S1b. *MTHFR* A1298C percentage of polymorphism per control and IHD case groups. Figure S2a. Forest plot for meta-analysis of *MTHFR* 677 polymorphism by TT genotype, countries of Caucasian with risks >1. Figure S2b. Forest plot for meta-analysis of *MTHFR* 677 polymorphism by TT genotype, countries with risks <1. Figure S2c. Forest plot for meta-analysis of *MTHFR* 677 polymorphism by TT genotype, countries with risks varied around 1. Figure S3a. Geographic information maps for percentages of *MTHFR* 677 TT genotype per control and IHD case groups, and its association with IHD risks. Figure S3b. Geographic information maps for percentages of *MTHFR* 677 TT plus CT genotypes per control and ischemic heart disease (IHD) case groups, and their associations with IHD risks.
