# Supplementary material for: A Meta-Prediction of Methylenetetrahydrofolate-Reductase Polymorphisms and Air Pollution Increased the Risk of Ischemic Heart Diseases Worldwide
Source: Int J Environ Res Public Health. 2018 Jul 10;15(7):1453. doi: 10.3390/ijerph15071453 (PMC6068673; doi:10.3390/ijerph15071453)
Supplement: Supplementary file 1 [file ijerph-15-01453-s001.pdf]

Supplementary Table S1. Summary of participant characteristics and *MTHFR* 677 and 1298 loci distributions for studies on ischemic heart disease (IHD) included in this review, by geographic location (112 articles, 123 study groups based on counts available from both control and IHD groups).

First Author (Reference number[1])	Year	Ethnicity – Country	Gender	*MTHFR* 677									*MTHFR* 1298							Quality Score[2]
				Cases, n (%)				Controls, n (%)					Cases, n (%)			Controls, n (%)				
				Types	CC	CT	TT	SOC	CC	CT	TT	HWE[3]	AA	AC	CC	AA	AC	CC	HWE	
**Oceania**																				
Wilcken (33)	1996	Caucasian Australia	Both	1	186 (40.8)	217 (47.6)	53 (11.6)	1	88 (39.1)	113 (50.2)	24 (10.7)	Yes								21 (8, 8, 5)
**Europe**																				
Trifonova (34)	2012	Caucasian Russia	Both	1	73 (51.8)	57 (40.4)	11 (7.8)	2	69 (54.8)	49 (38.9)	8 (6.3)	Yes	68 (48.2)	59 (41.8)	14 (9.9)	50 (39.7)	61 (48.4)	15 (11.9)	Yes	19 (8, 6, 5)
Spiridonova (35)	2001	Caucasian Russia	Male	1	44 (46.8)	41 (43.6)	9 (9.6)	2	64 (52.5)	48 (39.3)	10 (8.2)	Yes								17 (9, 4, 4)
Sarecka-Hujar (36)	2012	Caucasian Poland	Both	1	65 (42.8)	72 (47.4)	15 (9.9)	2	59 (48.8)	56 (46.3)	6 (5.0)	Yes								21 (7, 8, 6)
Sarecka-Hujar (37)	2008	Caucasian Poland	Female	1	23 (39.7)	29 (50)	6 (10.3)	2	25 (52.1)	21 (43.8)	2 (4.2)	Yes								23 (10, 7, 6)
		Caucasian Poland	Male	1	52 (43.7)	56 (47.1)	11 (9.2)	2	78 (50.6)	68 (44.2)	8 (5.2)	Yes								23 (10, 7, 6)
Strauss (38)	2005	Caucasian Poland	Male	1	82 (47.1)	74 (42.5)	18 (10.3)	1	58 (49.6)	45 (38.5)	14 (12.0)	Yes	86 (49.4)	66 (37.9)	22 (12.6)	59 (50.4)	50 (42.7)	8 (6.8)	Yes	22 (9, 7, 6)
Kadziela (39)	2003	Caucasian Poland	Both	1	58 (48.3)	51 (42.5)	11 (9.2)	2	46 (43.4)	49 (46.2)	11 (10.4)	Yes								17 (7, 5, 5)
Zak (40)	2003	Caucasian Poland	Both	1	29 (43.9)	31 (47.0)	6 (9.1)	2	47 (42.3)	56 (50.5)	8 (7.2)	Yes								16 (6, 5, 5)
Szczeklik (41)	2001	Caucasian Poland	Male	1	105 (65.2)	39 (24.2)	17 (10.6)	2	118 (55.9)	75 (35.5)	18 (8.5)	Yes	82 (50.9)	61 (37.9)	18 (11.2)	135 (64.0)	68 (32.2)	8 (3.8)	Yes	22 (10, 7, 5)
Gorqcy (42)	1999	Caucasian Poland	Both	2	46 (46.0)	45 (45.0)	9 (9.0)	3	39 (39.0)	50 (50.0)	11 (11.0)	Yes								16 (5, 6, 5)
Raslova (43)	2001	Caucasian Slovak	Both	2	22 (37.9)	29 (50.0)	7 (12.1)	3	36 (52.2)	29 (42.0)	4 (5.8)	Yes								22 (8, 8, 6)
Mehlig (44)	2013	Caucasian Sweden	Both	2	611 (53.1)	458 (39.8)	81 (7.0)	3	914 (52.1)	706 (40.3)	133 (7.6)	Yes								20 (8, 8, 4)
Thogersen (45)	2001	Caucasian Sweden	Both	2	32 (46.4)	32 (46.4)	5 (7.2)	3	71 (55.0)	51 (39.5)	7 (5.4)	Yes								23 (9, 8, 6)
Balogh (46)	2012	Caucasian Hungary	Both	1	276 (42.3)	312 (47.8)	65 (10.0)	2	137 (45.4)	13 (44.0)	32 (10.6)	Yes								21 (7, 8, 6)
Benes (47)	2001	Caucasian Czech	Both	1	55 (34.8)	80 (50.6)	23 (14.6)	1	86 (41.1)	106 (50.7)	17 (8.1)	No								22 (9, 7, 6)
Zuntar (48)	2003	Caucasian Croatia	Both	3c	114 (46.2)	111 (44.9)	22 (8.9)	1	134 (45.0)	146 (49.0)	18 (6.0)	Yes								15 (4, 7, 4)
Kolling (49)	2004	Caucasian Germany	Both	1	915 (43.1)	955 (45.0)	251 (11.8)	2	266 (43.1)	283 (45.9)	68 (11.0)	Yes	973 (45.9)	905 (42.7)	243 (11.5)	281 (45.5)	284 (46.0)	52 (8.4)	Yes	26 (9, 11, 6)
Blankenberg (50)	2002	Caucasian Germany	Both	1	370 (41.4)	417 (46.7)	106 (11.9)	2	143 (44.7)	143 (44.7)	34 (10.6)	Yes								18 (7, 6, 5)

First Author (Reference number[1])	Year	Ethnicity – Country	Gender	*MTHFR* 677									*MTHFR* 1298							Quality Score[2]
				Cases, n (%)				Controls, n (%)					Cases, n (%)			Controls, n (%)				
				Types	CC	CT	TT	SOC	CC	CT	TT	HWE[3]	AA	AC	CC	AA	AC	CC	HWE	
Rothenbacher (51)	2002	Caucasian Germany	Male	1	135 (50.6)	104 (39.0)	28 (10.5)	1	165 (46.0)	157 (43.7)	37 (10.3)	Yes	116 (43.4)	124 (46.4)	27 (10.2)	143 (39.9)	172 (48.0)	43 (12.0)	Yes	23 (8, 9, 6)
		Caucasian Germany	Female	1	20 (44.4)	22 (48.9)	3 (6.7)	1	54 (45.0)	53 (44.2)	13 (10.8)	Yes	17 (37.8)	25 (55.6)	3 (6.7)	51 (42.5)	46 (38.3)	23 (19.2)	No	
Meisel (52)	2001	Caucasian Germany	Both	1	458 (46.7)	442 (45.1)	81 (8.3)	2	443 (45.2)	442 (45.1)	96 (9.8)	Yes	430 (43.8)	458 (46.7)	93 (9.5)	433 (44.1)	442 (45.2)	105 (10.7)	Yes	22 (7, 10, 5)
Reinhardt (53)	1998	Caucasian Germany	Both	1	91 (50.6)	66 (36.7)	23 (12.8)	2	49 (47.1)	46 (44.2)	9 (8.7)	Yes								20 (8, 8, 4)
Rossi (54)	2006	Caucasian Italy	Both	3a	42 (33.9)	53 (42.7)	29 (23.4)	2	23 (31.5)	33 (45.2)	17 (23.3)	Yes								21 (9, 7, 5)
Girelli (55)	2003	Caucasian Italy	Both	1	146 (33.7)	217 (50.1)	70 (16.2)	2	75 (33.8)	105 (47.3)	42 (18.9)	Yes								20 (7, 8, 5)
Ardissino (56)	1999	Caucasian Italy	Both	2	68 (34.0)	97 (48.5)	35 (17.5)	2	60 (30.0)	102 (51.0)	38 (19.0)	Yes								20 (8, 7, 5)
Tanis (57)	2004	Caucasian Netherlands	Female	2	78 (43.1)	81 (44.8)	22 (12.2)	2	280 (46.6)	262 (43.6)	59 (9.8)	Yes								22 (9, 8, 5)
Verhoeff (58)	1998	Caucasian Netherlands	Both	3a	137 (53.3)	93 (36.2)	27 (10.5)	1	129 (47.4)	105 (38.6)	38 (14.0)	No								21 (8, 7, 6)
Kluijtmans (59)	1997	Caucasian Netherlands	Male	1	337 (45.9)	328 (44.6)	70 (9.5)	1	617 (49.4)	527 (42.2)	106 (8.5)	Yes								21 (7, 9, 5)
Verhoef (60)	1997	Caucasian Netherlands	Both	1	59 (45.0)	59 (45.0)	13 (9.9)	1	45 (45.0)	48 (48.0)	7 (7.0)	Yes								20 (8, 7, 5)
Kluijtmans (61)	1996	Caucasian Netherlands	Both	3a	30 (50.0)	21 (35.0)	9 (15.0)	2	63 (56.8)	42 (37.8)	6 (5.4)	Yes								19 (7, 7, 5)
Todesco (62)	1999	Caucasian Switzerland	Both	3a	30 (40.0)	34 (45.3)	11 (14.7)	1	103 (46.0)	93 (41.5)	28 (12.5)	Yes								23 (9, 8, 6)
Guéant-Rodrigue z (63)	2005	Caucasian France	Both	1	210 (39.6)	247 (46.6)	73 (13.8)	1	105 (42.3)	113 (45.6)	30 (12.1)	Yes	224 (42.3)	241 (45.5)	65 (12.3)	108 (43.5)	111 (44.8)	29 (11.7)	Yes	21 (9, 7, 5)
Pinto (64)	2001	Caucasian Spain	Male	1	31 (40.8)	34 (44.7)	11 (14.5)	1	39 (41.1)	43 (45.3)	13 (13.7)	Yes								19 (6, 8, 5)
Virgos (65)	2000	Caucasian Spain	Male	2	34 (47.2)	33 (45.8)	5 (6.9)	1	27 (37.5)	31 (43.1)	14 (19.4)	Yes								21 (7, 8, 6)
Freitas (66)	2008	Caucasian Portugal	Both	1	130 (43.6)	136 (45.6)	32 (10.7)	2	262 (51.4)	200 (39.2)	48 (9.4)	Yes	158 (53.0)	123 (41.3)	17 (5.7)	222 (43.5)	259 (50.8)	29 (5.7)	Yes	22 (10, 7, 5)
Araujo (67)	2000	Caucasian Portugal	Both	1	74 (37.2)	103 (51.8)	22 (11.1)	1	10 (50.0)	8 (40.0)	2 (10.0)	Yes								18 (9, 6, 3)
Ferrer-Antunes (68)	1998	Caucasian Portugal	Both	2	54 (42.5)	59 (46.5)	14 (11.0)	1	71 (55.9)	51 (40.2)	5 (3.9)	Yes								15 (7, 4, 4)
Chambers (69)	2000	South Asian U.K.	Male	1	160 (71.4)	61 (27.2)	3 (1.3)	1	279 (73.2)	90 (23.6)	12 (3.1)	Yes								23 (8, 9, 6)
		Caucasian U.K.	Male	1	108 (47.0)	91 (39.6)	31 (13.5)	1	188 (44.3)	195 (46.0)	41 (9.7)	Yes								
Malik (70)	1998	Caucasian U.K.	Both	1	107 (40.1)	134 (50.2)	26 (9.7)	2	106 (45.5)	110 (47.2)	17 (7.3)	Yes								21 (9, 7, 5)
Adams (71)	1996	Caucasian U.K.	Both	2	133 (42.9)	145 (46.8)	32 (10.3)	1	96 (43.2)	97 (43.7)	29 (13.1)	Yes								24 (8, 11, 5)

First Author (Reference number[1])	Year	Ethnicity – Country	Gender	*MTHFR 677*									*MTHFR 1298*							Quality Score[2]
				Cases, n (%)				Controls, n (%)					Cases, n (%)			Controls, n (%)				
				Types	CC	CT	TT	SOC	CC	CT	TT	HWE[3]	AA	AC	CC	AA	AC	CC	HWE	
Gallagher (72)	1996	Caucasian Ireland	Both	3b	44 (39.6)	48 (43.2)	19 (17.1)	2	53 (50.5)	45 (42.9)	7 (6.7)	Yes								22 (9, 8, 5)
**North America**																				
Christensen (73)	1997	Caucasian Canada	Both	1	62 (40.8)	68 (44.7)	22 (14.5)	1	47 (38.8)	61 (50.4)	13 (10.7)	Yes								21 (8, 8, 5)
McCarthy (74)	2004	Caucasian U.S.	Both	1	224 (46.5)	180 (37.3)	78 (16.2)	1	191 (46.7)	172 (42.1)	46 (11.2)	Yes								23 (10, 8, 5)
Brilakis (75)	2003	Caucasian U.S.	Both	1	117 (43.2)	123 (45.4)	31 (11.4)	2	109 (46.8)	101 (43.3)	23 (9.9)	Yes								21 (8, 8, 5)
Tsai (76)	1999	Caucasian U.S.	Both	1	159 (42.3)	177 (47.1)	40 (10.6)	1	35 (42.7)	35 (42.7)	12 14.6)	Yes								21 (8, 7, 6)
Verhoef (77)	1998	Caucasian U.S.	Male	1	230 (46.0)	209 (41.8)	61 (12.2)	2	228 (45.6)	200 (40.0)	72 (14.4)	No								24 (8, 11, 5)
Anderson (78)	1997	Caucasian U.S.	Both	2	90 (45)	87 (43.5)	23 (11.5)	3	257 (46.4)	238 (43)	59 (10.6)	Yes								16 (5, 5, 6)
		Caucasian U.S.	Both	1	241 (47.3)	212 (41.6)	57 (11.2)	2	73 (43.5)	73 (43.5)	22 (13.1)	Yes								16 (5, 5, 6)
Brugada (79)	1997	Caucasian U.S.	Male	1	58 (51.8)	49 (43.8)	5 (4.5)	2	52 (46.4)	54 (48.2)	6 (5.4)	Yes								22 (9, 7, 6)
		Caucasian U.S.	Female	1	18 (41.9)	20 (46.5)	5 (11.6)	2	18 (41.9)	19 (44.2)	6 (14.0)	Yes								
Malinow (80)	1997	Caucasian U.S.	Both	1	40 (28.6)	83 (59.3)	17 (12.1)	2	49 (48.0)	45 (44.1)	8 (7.8)	Yes								21 (8, 8, 5)
Ma (81)	1996	Caucasian U.S.	Male	2	136 (46.4)	124 (42.3)	33 (11.3)	3	135 (46.6)	116 (40.0)	39 (13.4)	Yes								24 (7, 12, 5)
Hanson (82)	2001	Mixed U.S.	Both	3a	324 (42.0)	364 (47.2)	84 (10.9)	1	130 (39.5)	158 (48.0)	41 (12.5)	Yes	360 (46.6)	322 (41.7)	90 (11.7)	164 (49.8)	139 (42.2)	26 (7.9)	Yes	21 (8, 8, 5)
Dilley (83)	2001	Mixed U.S.	Both	1	91 (82.7)	17 (15.5)	2 (1.8)	1	153 (82.7)	28 (15.1)	4 (2.2)	Yes								22 (8, 9, 5)
Schwartz (84)	1997	Mixed U.S.	Female	2	28 (40.6)	34 (49.3)	7 (10.1)	1	154 (45.6)	141 (41.7)	43 (12.7)	Yes								23 (8, 9, 6)
**Central America**																				
Isordia-Salas (85)	2010	Hispanic Mexico	Both	2	38 (22.8)	75 (44.9)	54 (32.3)	1	42 (25.1)	78 (46.7)	47 (28.1)	Yes								19 (8, 6, 5)
Salazar-Sanchez (86)	2006	Hispanic Costa Rica	Both	2	48 (25.8)	90 (48.4)	48 (25.8)	1	59 (29.9)	79 (40.1)	59 (29.9)	No								20 (9, 6, 5)
**South America**																				
Biselli (87)	2009	Caucasian Brazil	Both	1	62 (35.4)	93 (53.1)	20 (11.4)	2	39 (36.1)	59 (54.6)	10 (9.3)	Yes	101 (57.7)	67 (38.3)	7 (4.0)	54 (50.0)	49 (45.4)	5 (4.6)	Yes	20 (8, 6, 6)
Lima (88)	2007	Caucasian Brazil	Both	1	9 (31.0)	18 (62.1)	2 (6.9)	2	13 (65.0)	6 (30.0)	1 (5.0)	Yes								23 (8, 8, 7)
Rios (89)	2007	African Brazil	Male	1	67 (63.2)	35 (33.0)	4 (3.8)	2	38 (64.4)	20 (33.9)	1 (1.7)	Yes								22 (9, 7, 6)
		African Brazil	Female	1	39 (69.9)	15 (26.8)	2 (3.6)	2	50 (70.4)	19 (26.8)	2 (2.8)	Yes								
		Caucasian	Male	1	108	82	22	2	45	28	1	Yes								

First Author (Reference number[1])	Year	Ethnicity – Country	Gender	*MTHFR* 677									*MTHFR* 1298							Quality Score[2]
				Cases, n (%)				Controls, n (%)					Cases, n (%)			Controls, n (%)				
				Types	CC	CT	TT	SOC	CC	CT	TT	HWE[3]	AA	AC	CC	AA	AC	CC	HWE	
		Brazil			(50.9)	(38.7)	(10.4)		(60.8)	(37.8)	(1.4)									
		Caucasian Brazil	Female	1	45 (40.9)	61 (55.5)	4 (3.6)	2	46 (54.8)	31 (36.9)	7 (8.3)	Yes								
Muniz (90)	2006	Caucasian Brazil	Both	1	57 (61.3)	28 (30.1)	8 (8.6)	1	68 (63.0)	31 (28.7)	9 (8.3)	Yes								16 (5, 6, 5)
Helfenstein (91)	2005	Caucasian Brazil	Both	2	24 (55.8)	14 (32.6)	5 (11.6)	3	26 (52)	20 (40)	4 (8)	Yes								23 (8, 8, 7)
		Caucasian Brazil	Both	2	21 (44.7)	21 (44.7)	5 (10.6)	3	26 (46.4)	24 (42.9)	6 (10.7)	Yes								
**East Asia**																				
Yamada (92)	2006	East Asian Japan	Both	2	375 (31.5)	570 (47.8)	247 (20.7)	1	804 (35.1)	1134 (49.5)	353 (15.4)	Yes								21 (9, 7, 5)
Shioji (93)	2004	East Asian Japan	Male	2	160 (34.7)	226 (49.0)	75 (16.3)	3	293 (34.7)	411 (48.6)	141 (16.7)	Yes								17 (7, 5, 5)
		East Asian Japan	Female	2	33 (47.1)	24 (34.3)	13 (18.6)	3	370 (37.0)	467 (46.7)	164 (16.4)	Yes								
Nakai (94)	2000	East Asian Japan	Male	2	93 (40.4)	95 (41.3)	42 (18.3)	1	81 (40.9)	96 (48.5)	21 (10.6)	Yes								21 (8, 8, 5)
Morita (95)	1998	East Asian Japan	Male	1	116 (32.6)	183 (51.4)	57 (16.0)	2	207 (42.4)	234 (48.0)	47 (9.6)	Yes								15 (7, 4, 4)
Ou (96)	1998	East Asian Japan	Both	1	69 (32.2)	84 (39.3)	61 (28.5)	2	110 (35.5)	158 (51.0)	42 (13.5)	Yes								20 (8, 7, 5)
Izumi (97)	1996	East Asian Japan	Both	1	90 (36.0)	110 (44.0)	50 (20.0)	2	74 (36.8)	102 (50.7)	25 (12.4)	Yes								16 (6, 6, 4)
Jang (98)	2002	East Asian South Korea	Male	1	49 (32.9)	77 (51.7)	23 (15.4)	1	67 (29.1)	115 (50.0)	48 (20.9)	Yes								21 (8, 7, 6)
Hong (99)	2001	East Asian South Korea	Both	1	40 (28.6)	74 (52.9)	26 (18.6)	1	37 (26.4)	78 (55.7)	25 (17.9)	Yes								20 (7, 7, 6)
Lin (100)	2008	00)East Asian Taiwan	Both	1	66 (54.4)	47 (38.8)	8 (6.6)	1	88 (56.8)	57 (36.8)	10 (6.5)	Yes								20 (8, 7, 5)
Kou (101)	2001	East Asian Taiwan	Both	1	29 (53.7)	19 (35.2)	6 (11.1)	2	35 (63.6)	18 (32.7)	2 (3.6)	Yes	36 (66.7)	16 (29.6)	2 (3.7)	36 (65.5)	16 (29.1)	3 (5.5)	Yes	15 (6, 6, 3)
Hsu (102)	2001	East Asian Taiwan	Both	3d	120 (55.0)	85 (39.0)	13 (6.0)	2	125 (57.3)	78 (35.8)	15 (6.9)	Yes								21 (9, 7, 5)
Chen (103)	2000	East Asian Taiwan	Both	1	33 (55.0)	23 (38.3)	4 (6.7)	2	62 (56.9)	42 (38.5)	5 (4.6)	Yes								16 (7, 5, 4)
Chao (104)	1999	East Asian Taiwan	Both	1	60 (51.7)	47 (40.5)	9 (7.8)	2	41 (53.9)	25 (32.9)	10 (13.2)	Yes								19 (8, 6, 5)
Chen (105)	2014	East Asian China	Both	1	98 (23.1)	209 (49.3)	117 (27.6)	2	141 (29.6)	221 (46.4)	114 (23.9)	Yes								25 (9, 11, 5)
Yu (106)	2013	East Asian China	Both	3c	401 (35.4)	523 (46.2)	209 (18.4)	2	422 (38.2)	542 (49.0)	142 (12.8)	Yes								22 (9, 7, 6)
Yang (107)	2011	East Asian China	Both	1	38 (18.1)	96 (45.7)	76 (36.2)	1	88 (35.3)	110 (44.2)	51 (20.5)	Yes								16 (5, 7, 4)

First Author (Reference number[1])	Year	Ethnicity – Country	Gender	*MTHFR 677*									*MTHFR 1298*							Quality Score[2]
				Cases, n (%)				Controls, n (%)					Cases, n (%)			Controls, n (%)				
				Types	CC	CT	TT	SOC	CC	CT	TT	HWE[3]	AA	AC	CC	AA	AC	CC	HWE	
Luo (108)	2007	East Asian China	Both	1	27 (25.7)	61 (58.1)	17 (16.2)	1	44 (35.2)	63 (50.4)	18 (14.4)	Yes								15 (4, 6, 5)
Chen (109)	2007	East Asian China	Both	1	44 (23.3)	108 (57.1)	37 (19.6)	1	61 (46.6)	47 (35.9)	23 (17.6)	Yes								21 (8, 7, 6)
Niu (110)	2005	East Asian China	Both	3	18 (31.0)	28 (48.3)	12 (20.7)	2	19 (42.2)	23 (51.1)	3 (6.7)	Yes								22 (9, 8, 5)
Mu (111)	2005	East Asian China	Both	2	12 (25.5)	27 (57.4)	8 (17.0)	3	27 (55.1)	19 (38.8)	3 (6.1)	Yes								19 (7, 7, 5)
Sun (112)	2005	East Asian China	Both	1	43 (34.1)	52 (41.3)	31 (24.6)	1	58 (56.9)	26 (25.5)	18 (17.6)	No								22 (9, 8, 5)
Jiang (113)	2004	East Asian China	Both	1	16 (20.5)	39 (50.0)	23 (29.5)	1	29 (29.0)	46 (46.0)	25 (25.0)	Yes								18 (6, 7, 5)
Fang (114)	2002	East Asian China	Both	1	34 (21.1)	80 (49.7)	47 (29.2)	1	44 (35.2)	60 (48.0)	21 (16.8)	Yes								21 (7, 8, 6)
Mao (115)	2002	East Asian China	Both	1	53 (17.8)	142 (47.7)	103 (34.6)	2	27 (19.9)	61 (44.9)	48 (35.3)	Yes								21 (8, 7, 6)
Yu (116)	2000	East Asian China	Both	1	10 (14.5)	36 (52.2)	23 (33.3)	1	26 (32.9)	43 (54.4)	10 (12.7)	Yes								22 (9, 7, 6)
Xu (117)	1999	East Asian China	Both	1	15 (22.4)	29 (43.3)	23 (34.3)	2	20 (45.5)	15 (34.1)	9 (20.5)	Yes								16 (6, 7, 3)
Li (118)	2010	East Asian China	Both	1	36 (31.6)	51 (44.7)	27 (23.7)	2	10 (32.3)	15 (48.4)	6 (19.4)	Yes								17 (9, 5, 3)
Li (119)	2005	East Asian China	Both	2	62 (38.5)	83 (51.6)	16 (9.9)	2	37 (50.0)	32 (43.2)	5 (6.8)	Yes								21 (9, 7, 5)
Gao (120)	2004	East Asian China	Both	2	22 (22.9)	48 (50.0)	26 (27.1)	2	40 (48.8)	32 (39.0)	10 (12.2)	Yes								18 (6, 7, 5)
Zhang (121)	2001	East Asian China	Both	1	32 (43.8)	33 (45.2)	8 (11.0)	1	37 (37.0)	47 (47.0)	16 (16.0)	Yes								19 (6, 7, 6)
Zheng (122)	2000	East Asian China	Both	3d	41 (56.9)	29 (40.3)	2 (2.8)	1	62 (50.8)	45 (36.9)	15 (12.3)	Yes								23 (9, 8, 6)
**South Asia**																				
Dogra (123)	2012	South Asian India	Both	3d	120 (65.2)	55 (29.9)	9 (4.9)	1	250 (71.4)	91 (26.0)	9 (2.6)	Yes								22 (8, 8, 6)
Gupta (124)	2012	South Asian India	Both	1	132 (66.3)	64 (32.2)	3 (1.5)	1	154 (77.0)	45 (22.5)	1 (0.5)	Yes								20 (6, 8, 6)
Kanth (125)	2011	South Asian India	Both	1	93 (93.0)	6 (6.0)	1 (1.0)	2	196 (98.0)	4 (2.0)	0 (0.0)	Yes								18 (6, 7, 5)
Dayakar (126)	2011	South Asian India	Both	2	115 (75.7)	35 (23.0)	2 (1.3)	1	159 (92.5)	8 (4.8)	0 (0.0)	Yes	80 (52.6)	56 (36.8)	16 (10.5)	139 (83.2)	27 (16.2)	1 (0.6)	Yes	17 (6, 6, 5)
Vijaya (127)	2011	South Asian India	Both	1	256 (73.1)	88 (25.1)	6 (1.7)	1	231 (82.5)	49 (17.5)	0 (0.0)	Yes								20 (7, 7, 6)
Dhar (128)	2010	South Asian India	Both	1	112 (51.6)	47 (21.7)	58 (26.7)	1	186 (72.9)	36 (14.1)	33 (12.9)	No								22 (9, 7, 6)
Angeline (129)	2007	South Asian India	Male	2	81 (81.0)	18 (18.0)	1 (1.0)	1	84 (84.0)	16 (16.0)	0 (0.0)	Yes	38 (38.0)	46 (46.0)	16 (16.0)	48 (48.0)	38 (380)	14 (14.0)	Yes	18 (7, 6, 5)
Mukherjee (130)	2002	South Asian India	Male	3a	122 (62.6)	73 (37.4)	0 (0.0)	2	97 (63.8)	51 (33.6)	4 (2.6)	Yes								20 (7, 7, 6)

First Author (Reference number[1])	Year	Ethnicity – Country	Gender	*MTHFR 677*									*MTHFR 1298*							Quality Score[2]
				Cases, n (%)				Controls, n (%)					Cases, n (%)			Controls, n (%)				
				Types	CC	CT	TT	SOC	CC	CT	TT	HWE[3]	AA	AC	CC	AA	AC	CC	HWE	
		South Asian India	Female	3a	33 (58.9)	23 (41.1)	0 (0.0)	2	40 (75.5)	12 (22.6)	1 (1.9)	Yes								
Iqbal (131)	2005	South Asian Pakistan	Both	2	279 (70.3)	110 (27.7)	8 (2.0)	1	161 (71.6)	57 (25.3)	7 (3.1)	Yes								21 (9, 7, 5)
**Middle-East**																				
Heidari (132)	2016	Mid-Eastern Iran	Both	1	54 (50.0)	36 (33.3)	18 (16.7)	1	61 (67.8)	27 (30.0)	2 (2.2)	Yes								20 (8, 7, 5)
Abu-Amero (133)	2003	Mid-Eastern Saudi Arabia	Both	1	350 (64.2)	175 (32.1)	20 (3.7)	1	451 (72.2)	161 (25.8)	13 (2.1)	Yes	247 (45.7)	253 (46.9)	40 (7.4)	246 (39.4)	322 (51.5)	57 (9.1)	No	15 (4, 7, 4)
Ilhan (134)	2008	Mid-Eastern Turkey	Both	1	52 (52.0)	44 (44.0)	4 (4.0)	1	72 (72.0)	26 (26.0)	2 (2.0)	Yes								20 (9, 6, 5)
Gulec (135)	2001	Mid-Eastern Turkey	Male	2	42 (43.8)	39 (40.6)	15 (15.6)	1	60 (60.0)	35 (35.0)	5 (5.0)	Yes								21 (9, 6, 6)
Tokgozoglu (136)	1999	Mid-Eastern Turkey	Both	1	69 (45.7)	71 (47.0)	11 (7.3)	2	47 (51.6)	39 (42.9)	5 (5.5)	Yes								16 (5, 5, 6)
Almawi (137)	2004	Mid-Eastern Lebanon	Both	3d	27 (28.1)	39 (40.6)	30 (31.3)	1	220 (54.4)	166 (41.1)	18 (4.5)	Yes								18 (8, 5, 5)
Mager (138)	1999	Mid-Eastern Israel	Both	1	52 (30.8)	85 (50.3)	32 (18.9)	1	130 (41.5)	139 (44.4)	44 (14.1)	Yes								22 (9, 7, 6)
**Africa**																				
El-Sammak (139)	2004	African Egypt	Male	2	22 (44.0)	22 (44.0)	6 (12.0)	1	22 (44.0)	24 (48.0)	4 (8.0)	Yes								28 (11, 12, 5)
Ghazouani (140)	2009	African Tunisia	Both	1	157 (44.6)	149 (42.3)	46 (13.1)	1	247 (63.3)	123 (31.5)	20 (5.1)	Yes	208 (59.1)	124 (35.2)	20 (5.7)	237 (60.8)	135 (34.6)	18 (4.6)	Yes	21 (9, 7, 5)
Kerkeni (141)	2006	African Tunisia	Both	1	49 (49.0)	35 (35.0)	16 (16.0)	1	58 (48.3)	55 (45.8)	7 (5.8)	Yes	58 (58.0)	31 (31.0)	11 (11.0)	68 (56.7)	43 (35.8)	9 (7.5)	Yes	23 (9, 8, 6)
Bennouar (142)	2007	African Morocco	Both	1	101 (48.1)	78 (37.1)	31 (14.8)	2	113 (59.5)	61 (32.1)	16 (8.4)	Yes								23 (9, 8, 6)
Ramkaran (143)	2015	South Asian South Africa	Male	1	79 (74.5)	25 (23.6)	2 (1.9)	2	86 (86.0)	14 (14.0)	0 (0.0)	Yes								21 (9, 7, 5)
Ranjith (144)	2003	South Asian South Africa	Both	2	166 (85.1)	29 (14.9)	0 (0.0)	1	238 (79.3)	58 (19.3)	4 (1.3)	Yes	75 (38.5)	78 (40.0)	42 (21.5)	102 (34.0)	152 (50.7)	46 (15.3)	Yes	20 (7, 8, 5)

Note. Types of ischemic heart disease (IHD): 1 = coronary artery disease (CAD), 2 = myocardial infarction (MI), 3 = mixed IHDs and others; 3a = coronary heart disease; 3b = premature cardiovascular disease; 3c = peripheral vascular atherosclerotic disease; 3d = arterial and venous thrombosis; Sources of controls (SOC): 1 = healthy adults, 2 = adults without CAD, 3 = adults without MI; NA: Not available; [1]Reference numbers refer to the Reference List that follows this table; [2]Quality score ranges: Total score 0–29; external validity 0–11; internal validity 0–12; report quality 0–6; [3]HWE = Hardy-Weinberg equilibrium, updated report based on calculation using the formula available at http://www.koonec.com/k-blog/2010/06/20/hardy-weinberg-equilibrium-calculator.

Studies included in the meta-analysis

30 Meta-analysis

1. Bentley P, Peck G, Smeeth L, Whittaker J, Sharma P. Causal relationship of susceptibility genes to ischemic stroke: comparison to ischemic heart disease and biochemical determinants. PLoS One. 2010; 5(2):e9136. https://doi.org/10.1371/journal.pone.0009136 PMID: 20161734
2. Brattstrom L, Wilcken DE, Ohrvik J, Brudin L. Common methylenetetrahydrofolate reductase gene mutation leads to hyperhomocysteinemia but not to vascular disease: the result of a meta-analysis. Circulation. 1998; 98(23):2520-6. PMID: 9843457
3. Chen L, Liu L, Hong K, Hu J, Cheng X. Three genetic polymorphisms of homocysteine-metabolizing enzymes and risk of coronary heart disease: a meta-analysis based on 23 case-control studies. DNA Cell Biol. 2012; 31(2):238-49. https://doi.org/10.1089/dna.2011.1281 PMID: 21780915
4. Clarke R, Bennett DA, Parish S, Verhoef P, Dotsch-Klerk M, Lathrop M, et al. Homocysteine and coronary heart disease: meta-analysis of *MTHFR* case-control studies, avoiding publication bias. PLoS Med. 2012; 9(2):e1001177. https://doi.org/10.1371/journal.pmed.1001177 PMID: 22363213
5. Clarke R, Lewington S, Landray M. Homocysteine, renal function, and risk of cardiovascular disease. Kidney Int Suppl. 2003; (84):S131-3. https://doi.org/10.1046/j.1523-1755.63.s84.7.x PMID: 12694328
6. Drenos F, Whittaker JC, Humphries SE. The use of meta-analysis risk estimates for candidate genes in combination to predict coronary heart disease risk. Annals of human genetics. 2007; 71(Pt 5):611-9. https://doi.org/10.1111/j.1469-1809.2007.00359.x PMID: 17403027
7. Ganesh SK, Tragante V, Guo W, Guo Y, Lanktree MB, Smith EN, et al. Loci influencing blood pressure identified using a cardiovascular gene-centric array. Human molecular genetics. 2013; 22(8):1663-78. https://doi.org/10.1093/hmg/dds555 PMID: 23303523
8. Gil Nunez AC. [Homocysteine: vascular risk factor?]. Neurologia. 2004; 19(1):1-4. PMID: 14762726
9. Hou X, Chen X, Shi J. Genetic polymorphism of *MTHFR* C677T and premature coronary artery disease susceptibility: A meta-analysis. Gene. 2015; 565(1):39-44. https://doi.org/10.1016/j.gene.2015.03.062 PMID: 25839940
10. Jee SH, Beaty TH, Suh I, Yoon Y, Appel LJ. The methylenetetrahydrofolate reductase gene is associated with increased cardiovascular risk in Japan, but not in other populations. Atherosclerosis. 2000; 153(1):161-8. PMID: 11058711
11. Kim RJ, Becker RC. Association between factor V Leiden, prothrombin G20210A, and methylenetetrahydrofolate reductase C677T mutations and events of the arterial circulatory system: a meta-analysis of published studies. Am Heart J. 2003; 146(6):948-57. https://doi.org/10.1016/s0002-8703(03)00519-2 PMID: 14660985
12. Klerk M, Verhoef P, Clarke R, Blom HJ, Kok FJ, Schouten EG. *MTHFR* 677C-->T polymorphism and risk of coronary heart disease: a meta-analysis. JAMA. 2002; 288(16):2023-31. PMID: 12387655
13. Kluijtmans LA, Kastelein JJ, Lindemans J, Boers GH, Heil SG, Bruschke AV, et al. Thermolabile methylenetetrahydrofolate reductase in coronary artery disease. Circulation. 1997; 96(8):2573-7. PMID: 9355896
14. Kluijtmans LA, Whitehead AS. Methylenetetrahydrofolate reductase genotypes and predisposition to atherothrombotic disease; evidence that all three *MTHFR* C677T genotypes confer different levels of risk. Eur Heart J. 2001; 22(4):294-9. https://doi.org/10.1053/euhj.2000.2239 PMID: 11161947
15. Lewis SJ, Ebrahim S, Davey Smith G. Meta-analysis of *MTHFR* 677C->T polymorphism and coronary heart disease: does totality of evidence support causal role for homocysteine and preventive potential of folate? BMJ. 2005; 331(7524):1053. https://doi.org/10.1136/bmj.38611.658947.55 PMID: 16216822
16. Li YY. Methylenetetrahydrofolate reductase C677T gene polymorphism and coronary artery disease in a Chinese Han population: a meta-analysis. Metab Clin Exp. 2012; 61(6):846-52. https://doi.org/10.1016/j.metabol.2011.10.013 PMID: 22146089
17. Luo H, Liu B, Hu J, Wang X, Zhan S, Kong W. Hyperhomocysteinemia and methylenetetrahydrofolate reductase polymorphism in cervical artery dissection: a meta-analysis. Cerebrovasc Dis. 2014; 37(5):313-22. https://doi.org/10.1159/000360753 PMID: 24903192
18. Mager A. Methylenetetrahydrofolate reductase gene and coronary artery disease. Circulation. 2000; 101(16):E172-3. PMID: 10779474
19. Mehlig K, Leander K, de Faire U, Nyberg F, Berg C, Rosengren A, et al. The association between plasma homocysteine and coronary heart disease is modified by the *MTHFR* 677C>T polymorphism. Heart. 2013; 99(23):1761-5. https://doi.org/10.1136/heartjnl-2013-304460 PMID: 24014284

20. Minelli C, Thompson JR, Tobin MD, Abrams KR. An integrated approach to the meta-analysis of genetic association studies using Mendelian randomization. Am J Epidemiol. 2004; 160(5):445-52. https://doi.org/10.1093/aje/kwh228 PMID: 15321841
21. Pan F, Tian J, Zhang Y, Pan Y. Three genetic polymorphisms of homocysteine-metabolizing enzymes and risk of coronary heart disease: appraisal of a recent meta-analysis. DNA Cell Biol. 2012; 31(2):135-8. https://doi.org/10.1089/dna.2011.1481 PMID: 22087541
22. Samani NJ. Methylenetetrahydrofolate reductase mutation and coronary artery disease. Circulation. 1998; 98(25):2932-3. PMID: 9860798
23. Thompson JR, Minelli C, Abrams KR, Tobin MD, Riley RD. Meta-analysis of genetic studies using Mendelian randomization--a multivariate approach. Stat Med. 2005; 24(14):2241-54. https://doi.org/10.1002/sim.2100 PMID: 15887296
24. van Meurs JB, Pare G, Schwartz SM, Hazra A, Tanaka T, Vermeulen SH, et al. Common genetic loci influencing plasma homocysteine concentrations and their effect on risk of coronary artery disease. Am J Clin Nutr. 2013; 98(3):668-76. https://doi.org/10.3945/ajcn.112.044545 PMID: 23824729
25. Wald DS, Law M, Morris JK. Homocysteine and cardiovascular disease: evidence on causality from a meta-analysis. BMJ. 2002; 325(7374):1202. PMID: 12446535
26. Wald DS, Law M, Morris JK. The dose-response relation between serum homocysteine and cardiovascular disease: implications for treatment and screening. Eur J Cardiovasc Prev Rehabil. 2004; 11(3):250-3. PMID: 15179109
27. Wald DS, Morris JK, Wald NJ. Reconciling the evidence on serum homocysteine and ischaemic heart disease: a meta-analysis. PLoS One. 2011; 6(2):e16473. https://doi.org/10.1371/journal.pone.0016473 PMID: 21311765
28. Wu AH, Tsongalis GJ. Correlation of polymorphisms to coagulation and biochemical risk factors for cardiovascular diseases. Am J Cardiol. 2001; 87(12):1361-6. PMID: 11397354
29. Xuan C, Bai XY, Gao G, Yang Q, He GW. Association between polymorphism of methylenetetrahydrofolate reductase (*MTHFR*) C677T and risk of myocardial infarction: a meta-analysis for 8,140 cases and 10,522 controls. Arch Med Res. 2011; 42(8):677-85. https://doi.org/10.1016/j.arcmed.2011.11.009 PMID: 22154679
30. Zhao H, Shi Y. Association between methylenetetrahydrofolate reductase (*MTHFR*) C677T polymorphism and risk of myocardial infarction: need for clarification of data in a recent meta-analysis. Arch Med Res. 2012; 43(6):489; author reply 90. https://doi.org/10.1016/j.arcmed.2012.08.001 PMID: 22885090

2 Articles with duplicate use of data on genotype allele counts

31. Girelli D, Friso S, Trabetti E, Olivieri O, Russo C, Pessotto R, et al. Methylenetetrahydrofolate reductase C677T mutation, plasma homocysteine, and folate in subjects from northern Italy with or without angiographically documented severe coronary atherosclerotic disease: evidence for an important genetic-environmental interaction. Blood. 1998; 91(11):4158-63. PMID: 9596662
32. Morita H, Taguchi J, Kurihara H, Kitaoka M, Kaneda H, Kurihara Y, et al. Genetic polymorphism of 5,10-methylenetetrahydrofolate reductase (*MTHFR*) as a risk factor for coronary artery disease. Circulation. 1997; 95(8):2032-6. PMID: 9133512

112 articles with usable data by genes and outcomes

33. Wilcken DE, Wang XL, Sim AS, McCredie RM. Distribution in healthy and coronary populations of the methylenetetrahydrofolate reductase (*MTHFR*) *C677T* mutation. Arterioscler Thromb Vasc Biol. 1996; 16(7):878-82. PMID: 8673563
34. Trifonova EA, Spiridonova MG, Gabidulina TV, Urnov FD, Puzyrev VP, Stepanov VA. [Analysis of the *MTHFR* gene linkage disequilibrium structure and association of polymorphic gene variants with coronary atherosclerosis]. Genetika. 2012; 48(10):1207-20. PMID: 23270270
35. Spiridonova MG, Stepanov VA, Puzyrev VP, Karpov RS. The estimation of gametic disequilibrium between DNA markers in candidate genes for coronary artery disease (CAD) and the associations of gene complexes with risk factors for CAD. Int J Circumpolar Health. 2001; 60(2):222-7. PMID: 11507973
36. Sarecka-Hujar B, Zak I, Krauze J. The TT genotype of the *MTHFR* 677C > T polymorphism increases susceptibility to premature coronary artery disease in interaction with some of the traditional risk factors. Acta Medica (Hradec Kralove). 2012; 55(4):172-9. https://doi.org/10.14712/18059694.2015.42 PMID: 23631288

37. Sarecka-Hujar B, Zak I, Krauze J. Carrier-state of two or three polymorphic variants of *MTHFR*, IL-6 and ICAM1 genes increases the risk of coronary artery disease. Kardiol Pol. 2008; 66(12):1269-77. PMID: 19169973
38. Strauss E, Gluszek J, Pawlak AL. Age and hypertension related changes in genotypes of *MTHFR* 677C>T, 1298A>C and PON1 -108C>T SNPs in men with coronary artery disease (CAD). J Physiol Pharmacol. 2005; 56 Suppl 2:65-75. PMID: 16077191
39. Kadziela J, Janas J, Dzielinska Z, Szperl M, Gazdzik D, Chotkowska E, et al. The C677T mutation in methylenetetrahydrofolate reductase gene, plasma homocysteine concentration and the risk of coronary artery disease. Kardiol Pol. 2003; 59(7):17-26; discussion PMID: 14560345
40. Zak I, Niemiec P, Sarecka B, Balcerzyk A, Ciemniewski Z, Rudowska E, et al. Carrier-state of D allele in ACE gene insertion/deletion polymorphism is associated with coronary artery disease, in contrast to the C677-->T transition in the *MTHFR* gene. Acta Biochim Pol. 2003; 50(2):527-34. https://doi.org/035002527 PMID: 12833177
41. Szczeklik A, Sanak M, Jankowski M, Dropinski J, Czachor R, Musial J, et al. Mutation A1298C of methylenetetrahydrofolate reductase: risk for early coronary disease not associated with hyperhomocysteinemia. Am J Med Genet. 2001; 101(1):36-9. PMID: 11343335
42. Goracy I, Goracy J, Suliga M, Ciechanowicz A. [C677T gene polymorphism of methylenetetrahydrofolate reductase (*MTHFR*) in patients with myocardial infarction]. Pol Arch Med Wewn. 1999; 102(4):849-54. PMID: 10948708
43. Raslova K, Smolkova B, Vohnout B, Gasparovic J, Frohlich JJ. Risk factors for atherosclerosis in survivors of myocardial infarction and their spouses: comparison to controls without personal and family history of atherosclerosis. Metab Clin Exp. 2001; 50(1):24-9. PMID: 11172470
44. Mehlig K, Leander K, de Faire U, Nyberg F, Berg C, Rosengren A, et al. The association between plasma homocysteine and coronary heart disease is modified by the *MTHFR* 677C>T polymorphism. Heart. 2013; 99(23):1761-5. https://doi.org/10.1136/heartjnl-2013-304460 PMID: 24014284
45. Thogersen AM, Nilsson TK, Dahlen G, Jansson JH, Boman K, Huhtasaari F, et al. Homozygosity for the C677-->T mutation of 5,10-methylenetetrahydrofolate reductase and total plasma homocyst(e) ine are not associated with greater than normal risk of a first myocardial infarction in northern Sweden. Coron Artery Dis. 2001; 12(2):85-90. PMID: 11281306
46. Balogh E, Bereczky Z, Katona E, Koszegi Z, Edes I, Muszbek L, et al. Interaction between homocysteine and lipoprotein(a) increases the prevalence of coronary artery disease/myocardial infarction in women: a case-control study. Thromb Res. 2012; 129(2):133-8. https://doi.org/10.1016/j.thromres.2011.07.001 PMID: 21803402
47. Benes P, Kankova K, Muzik J, Groch L, Benedik J, Elbl L, et al. Methylenetetrahydrofolate reductase polymorphism, type II diabetes mellitus, coronary artery disease, and essential hypertension in the Czech population. Mol Genet Metab. 2001; 73(2):188-95. https://doi.org/10.1006/mgme.2001.3188 PMID: 11386855
48. Zuntar I, Topic E, Vukosavic D, Vukovic V, Demarin V, Begonja A, et al. Croatian population data for the C677T polymorphism in methylenetetrahydrofolate reductase: frequencies in healthy and atherosclerotic study groups. Clin Chim Acta. 2003; 335(1-2):95-100. PMID: 12927690
49. Kolling K, Ndrepepa G, Koch W, Braun S, Mehilli J, Schomig A, et al. Methylenetetrahydrofolate reductase gene C677T and A1298C polymorphisms, plasma homocysteine, folate, and vitamin B12 levels and the extent of coronary artery disease. Am J Cardiol. 2004; 93(10):1201-6. https://doi.org/10.1016/j.amjcard.2004.02.009 PMID: 15135689
50. Blankenberg S, Rupprecht HJ, Peetz D, Bickel C, Hofman KP, Tiret L, et al. [Homocysteine, methylenetetrahydrofolate reductase/C677T genotype and risk for coronary heart disease. The AtheroGene study]. Dtsch Med Wochenschr. 2002; 127(14):729-35. https://doi.org/10.1055/s-2002-24402 PMID: 11935469
51. Rothenbacher D, Fischer HG, Hoffmeister A, Hoffmann MM, Marz W, Bode G, et al. Homocysteine and methylenetetrahydrofolate reductase genotype: association with risk of coronary heart disease and relation to inflammatory, hemostatic, and lipid parameters. Atherosclerosis. 2002; 162(1):193-200. PMID: 11947914
52. Meisel C, Cascorbi I, Gerloff T, Stangl V, Laule M, Muller JM, et al. Identification of six methylenetetrahydrofolate reductase (*MTHFR*) genotypes resulting from common polymorphisms: impact on plasma homocysteine levels and development of coronary artery disease. Atherosclerosis. 2001; 154(3):651-8. PMID: 11257266
53. Reinhardt D, Sigusch HH, Vogt SF, Farker K, Muller S, Hoffmann A. Absence of association between a common mutation in the methylenetetrahydrofolate reductase gene and the risk of coronary artery disease. Eur J Clin Invest. 1998; 28(1):20-3. PMID: 9502183
54. Rossi GP, Maiolino G, Seccia TM, Burlina A, Zavattiero S, Cesari M, et al. Hyperhomocysteinemia predicts total and cardiovascular mortality in high-risk women. J Hypertens. 2006; 24(5):851-9.

https://doi.org/10.1097/01.hjh.0000222754.75196.5c PMID: 16612246
55. Girelli D, Martinelli N, Pizzolo F, Friso S, Olivieri O, Stranieri C, et al. The interaction between *MTHFR* 677 C-->T genotype and folate status is a determinant of coronary atherosclerosis risk. J Nutr. 2003; 133(5):1281-5. PMID: 12730410
56. Ardissino D, Mannucci PM, Merlini PA, Duca F, Fetiveau R, Tagliabue L, et al. Prothrombotic genetic risk factors in young survivors of myocardial infarction. Blood. 1999; 94(1):46-51. PMID: 10381497
57. Tanis BC, Blom HJ, Bloemenkamp DG, van den Bosch MA, Algra A, van der Graaf Y, et al. Folate, homocysteine levels, methylenetetrahydrofolate reductase (*MTHFR*) 677C --> T variant, and the risk of myocardial infarction in young women: effect of female hormones on homocysteine levels. J Thromb Haemost. 2004; 2(1):35-41. PMID: 14717963
58. Verhoeff BJ, Trip MD, Prins MH, Kastelein JJ, Reitsma PH. The effect of a common methylenetetrahydrofolate reductase mutation on levels of homocysteine, folate, vitamin B12 and on the risk of premature atherosclerosis. Atherosclerosis. 1998; 141(1):161-6. PMID: 9863549
59. Kluijtmans LA, Kastelein JJ, Lindemans J, Boers GH, Heil SG, Bruschke AV, et al. Thermolabile methylenetetrahydrofolate reductase in coronary artery disease. Circulation. 1997; 96(8):2573-7. PMID: 9355896
60. Verhoef P, Kok FJ, Kluijtmans LA, Blom HJ, Refsum H, Ueland PM, et al. The 677C-->T mutation in the methylenetetrahydrofolate reductase gene: associations with plasma total homocysteine levels and risk of coronary atherosclerotic disease. Atherosclerosis. 1997; 132(1):105-13. PMID: 9247365
61. Kluijtmans LA, van den Heuvel LP, Boers GH, Frosst P, Stevens EM, van Oost BA, et al. Molecular genetic analysis in mild hyperhomocysteinemia: a common mutation in the methylenetetrahydrofolate reductase gene is a genetic risk factor for cardiovascular disease. Am J Hum Genet. 1996; 58(1):35-41. PMID: 8554066
62. Todesco L, Angst C, Litynski P, Loehrer F, Fowler B, Haefeli WE. Methylenetetrahydrofolate reductase polymorphism, plasma homocysteine and age. Eur J Clin Invest. 1999; 29(12):1003-9. PMID: 10583447
63. Gueant-Rodriguez RM, Juilliere Y, Candito M, Adjalla CE, Gibelin P, Herbeth B, et al. Association of MTRRA66G polymorphism (but not of *MTHFR* C677T and A1298C, MTRA2756G, TCN C776G) with homocysteine and coronary artery disease in the French population. Thromb Haemost. 2005; 94(3):510-5. https://doi.org/10.1160/th05-04-0262 PMID: 16268464
64. Pinto X, Vilaseca MA, Garcia-Giralt N, Ferrer I, Pala M, Meco JF, et al. Homocysteine and the *MTHFR* 677C-->T allele in premature coronary artery disease. Case control and family studies. Eur J Clin Invest. 2001; 31(1):24-30. PMID: 11168435
65. Virgos C, Joven J, Simo JM, Vilella E, Camps J, Arcelus R, et al. Homocyst(e)ine and the C677T mutation of methylenetetrahydrofolate reductase in survivors of premature myocardial infarction. Clin Biochem. 2000; 33(6):509-12. PMID: 11074245
66. Freitas AI, Mendonca I, Guerra G, Brion M, Reis RP, Carracedo A, et al. Methylenetetrahydrofolate reductase gene, homocysteine and coronary artery disease: the A1298C polymorphism does matter. Inferences from a case study (Madeira, Portugal). Thromb Res. 2008; 122(5):648-56. https://doi.org/10.1016/j.thromres.2008.02.005 PMID: 18384842
67. Araujo F, Lopes M, Goncalves L, Maciel MJ, Cunha-Ribeiro LM. Hyperhomocysteinemia, *MTHFR* C677T genotype and low folate levels: a risk combination for acute coronary disease in a Portuguese population. Thromb Haemost. 2000; 83(3):517-8. PMID: 10744169
68. Ferrer-Antunes C, Palmeiro A, Morais J, Lourenco M, Freitas M, Providencia L. The mutation C677T in the methylene tetrahydrofolate reductase gene as a risk factor for myocardial infarction in the Portuguese population. T Thromb Haemost. 1998; 80(3):521-2. PMID: 9759639
69. Chambers JC, Ireland H, Thompson E, Reilly P, Obeid OA, Refsum H, et al. Methylenetetrahydrofolate reductase 677 C-->T mutation and coronary heart disease risk in UK Indian Asians. Arterioscler Thromb Vasc Biol. 2000; 20(11):2448-52. PMID: 11073851
70. Malik NM, Syrris P, Schwartzman R, Kaski JC, Crossman DC, Francis SE, et al. Methylenetetrahydrofolate reductase polymorphism (C-677T) and coronary artery disease. Clin Sci (Lond). 1998; 95(3):311-5. PMID: 9730850
71. Adams M, Smith PD, Martin D, Thompson JR, Lodwick D, Samani NJ. Genetic analysis of thermolabile methylenetetrahydrofolate reductase as a risk factor for myocardial infarction. QJM. 1996; 89(6):437-44. PMID: 8758047
72. Gallagher PM, Meleady R, Shields DC, Tan KS, McMaster D, Rozen R, et al. Homocysteine and risk of premature coronary heart disease. Evidence for a common gene mutation. Circulation. 1996; 94(9):2154-8. PMID: 8901666
73. Christensen B, Frosst P, Lussier-Cacan S, Selhub J, Goyette P, Rosenblatt DS, et al. Correlation of a common

mutation in the methylenetetrahydrofolate reductase gene with plasma homocysteine in patients with premature coronary artery disease. Arterioscler Thromb Vasc Biol. 1997; 17(3):569-73. PMID: 9102178
74. McCarthy JJ, Parker A, Salem R, Moliterno DJ, Wang Q, Plow EF, et al. Large scale association analysis for identification of genes underlying premature coronary heart disease: cumulative perspective from analysis of 111 candidate genes. J Med Genet. 2004; 41(5):334-41. PMID: 15121769
75. Brilakis ES, Berger PB, Ballman KV, Rozen R. Methylenetetrahydrofolate reductase (*MTHFR*) 677C>T and methionine synthase reductase (MTRR) 66A>G polymorphisms: association with serum homocysteine and angiographic coronary artery disease in the era of flour products fortified with folic acid. Atherosclerosis. 2003; 168(2):315-22. PMID: 12801615
76. Tsai MY, Welge BG, Hanson NQ, Bignell MK, Vessey J, Schwichtenberg K, et al. Genetic causes of mild hyperhomocysteinemia in patients with premature occlusive coronary artery diseases. Atherosclerosis. 1999; 143(1):163-70. PMID: 10208491
77. Verhoef P, Rimm EB, Hunter DJ, Chen J, Willett WC, Kelsey K, et al. A common mutation in the methylenetetrahydrofolate reductase gene and risk of coronary heart disease: results among U.S. men. J Am Coll Cardiol. 1998; 32(2):353-9. PMID: 9708460
78. Anderson JL, King GJ, Thomson MJ, Todd M, Bair TL, Muhlestein JB, et al. A mutation in the methylenetetrahydrofolate reductase gene is not associated with increased risk for coronary artery disease or myocardial infarction. J J Am Coll Cardiol. 1997; 30(5):1206-11. PMID: 9350916
79. Brugada R, Marian AJ. A common mutation in methylenetetrahydrofolate reductase gene is not a major risk of coronary artery disease or myocardial infarction. Atherosclerosis. 1997; 128(1):107-12. PMID: 9051203
80. Malinow MR, Nieto FJ, Kruger WD, Duell PB, Hess DL, Gluckman RA, et al. The effects of folic acid supplementation on plasma total homocysteine are modulated by multivitamin use and methylenetetrahydrofolate reductase genotypes. Arterioscler Thromb Vasc Biol. 1997; 17(6):1157-62. PMID: 9194768
81. Ma J, Stampfer MJ, Hennekens CH, Frosst P, Selhub J, Horsford J, et al. Methylenetetrahydrofolate reductase polymorphism, plasma folate, homocysteine, and risk of myocardial infarction in US physicians. Circulation. 1996; 94(10):2410-6. PMID: 8921781
82. Hanson NQ, Aras O, Yang F, Tsai MY. C677T and A1298C polymorphisms of the methylenetetrahydrofolate reductase gene: incidence and effect of combined genotypes on plasma fasting and post-methionine load homocysteine in vascular disease. Clin Chem. 2001; 47(4):661-6. PMID: 11274015
83. Dilley A, Hooper WC, El-Jamil M, Renshaw M, Wenger NK, Evatt BL. Mutations in the genes regulating methylene tetrahydrofolate reductase (*MTHFR* C-->T677) and cystathione beta-synthase (CBS G-->A919, CBS T-->c833) are not associated with myocardial infarction in African Americans. Thromb Res. 2001; 103(2):109-15. PMID: 11457468
84. Schwartz SM, Siscovick DS, Malinow MR, Rosendaal FR, Beverly RK, Hess DL, et al. Myocardial infarction in young women in relation to plasma total homocysteine, folate, and a common variant in the methylenetetrahydrofolate reductase gene. Circulation. 1997; 96(2):412-7. PMID: 9244205
85. Isordia-Salas I, Trejo-Aguilar A, Valades-Mejia MG, Santiago-German D, Leanos-Miranda A, Mendoza-Valdez L, et al. C677T polymorphism of the 5,10 *MTHFR* gene in young Mexican subjects with ST-elevation myocardial infarction. Arch Med Res. 2010; 41(4):246-50. https://doi.org/10.1016/j.arcmed.2010.04.008 PMID: 20637366
86. Salazar-Sanchez L, Chaves L, Cartin M, Schuster G, Wulff K, Schroder W, et al. Common polymorphisms and cardiovascular factors in patients with myocardial infarction of Costa Rica. Rev Biol Trop. 2006; 54(1):1-11. PMID: 18457169
87. Biselli PM, Guerzoni AR, Goloni-Bertollo EM, Godoy MF, Abou-Chahla JA, Pavarino-Bertelli EC. [*MTHFR* genetic variability on coronary artery disease development]. Rev Assoc Med Bras. 2009; 55(3):274-8. PMID: 19629345
88. Lima LM, Carvalho M, Fernandes AP, Sabino Ade P, Loures-Vale AA, da Fonseca Neto CP, et al. Homocysteine and methylenetetrahydrofolate reductase in subjects undergoing coronary angiography. Arq Bras Cardiol. 2007; 88(2):167-72. PMID: 17384833
89. Rios DL, D'Onofrio LO, Carvalho HG, Santos-Filho A, Galvao-Castro B. Sex-specific effect of the thermolabile C677T mutation in the methylenetetrahydrofolate reductase gene on angiographically assessed coronary artery disease in Brazilians. Hum Biol. 2007; 79(4):453-61. https://doi.org/10.1353/hub.2007.0053 PMID: 18075008
90. Muniz MT, Siqueira ER, Fonseca RA, D'Almeida V, Hotta JK, dos Santos JE, et al. [Evaluation of *MTHFR* C677T gene polymorphism and homocysteine level in coronary atherosclerotic disease]. Arq Bras Endocrinol Metabol.

2006; 50(6):1059-65. PMID: 17221112
91. Helfenstein T, Fonseca FA, Relvas WG, Santos AO, Dabela ML, Matheus SC, et al. Prevalence of myocardial infarction is related to hyperhomocysteinemia but not influenced by C677T methylenetetrahydrofolate reductase and A2756G methionine synthase polymorphisms in diabetic and non-diabetic subjects. Clin Chim Acta. 2005; 355(1-2):165-72. https://doi.org/10.1016/j.cccn.2004.12.002 PMID: 15820491
92. Yamada Y, Matsuo H, Segawa T, Watanabe S, Kato K, Hibino T, et al. Assessment of genetic risk for myocardial infarction. Thromb Haemost. 2006; 96(2):220-7. PMID: 16894468
93. Shioji K, Kokubo Y, Goto Y, Nonogi H, Iwai N. An association analysis between genetic polymorphisms of matrix metalloproteinase-3 and methylenetetrahydrofolate reductase and myocardial infarction in Japanese. J Thromb Haemost. 2004; 2(3):527-8. PMID: 15009479
94. Nakai K, Fusazaki T, Suzuki T, Ohsawa M, Ogiu N, Kamata J, et al. Genetic polymorphism of 5,10-methylenetetrahydrofolate increases risk of myocardial infarction and is correlated to elevated levels of homocysteine in the Japanese general population. Coron Artery Dis. 2000; 11(1):47-51. PMID: 10715806
95. Morita H, Kurihara H, Taguchi J, Ohno M, Yazaki Y. ACE and *MTHFR* gene polymorphisms: genetic coronary risk factors relating to different aspects of pathophysiology. Thromb Haemost. 1998; 80(1):200-1. PMID: 9684811
96. Ou T, Yamakawa-Kobayashi K, Arinami T, Amemiya H, Fujiwara H, Kawata K, et al. Methylenetetrahydrofolate reductase and apolipoprotein E polymorphisms are independent risk factors for coronary heart disease in Japanese: a case-control study. Atherosclerosis. 1998; 137(1):23-8. PMID: 9568733
97. Izumi M, Iwai N, Ohmichi N, Nakamura Y, Shimoike H, Kinoshita M. Molecular variant of 5,10-methylenetetrahydrofolate reductase is a risk factor of ischemic heart disease in the Japanese population. Atherosclerosis. 1996; 121(2):293-4. PMID: 9125303
98. Jang Y, Park HY, Lee JH, Ryu HJ, Kim JY, Kim OY. A polymorphism of the methylenetetrahydrofolate reductase and methionine synthase gene in CAD patients: association with plasma folate, vitamin B_{12} and homocysteine. Nutr Res. 22(9):965-76. https://doi.org/10.1016/S0271-5317(02)00416-5
99. Hong SH, Song J, Kim JQ. Genetic variation of the methylenetetrahydrofolate reductase and cystathionine beta-synthase genes in Korean patients with coronary artery disease and a new polymorphism in intron 7. Mol Cell Probes. 2001; 15(2):119-23. https://doi.org/10.1006/mcpr.2001.0351 PMID: 11292330
100. Lin PT, Huang MC, Lee BJ, Cheng CH, Tsai TP, Huang YC. High plasma homocysteine is associated with the risk of coronary artery disease independent of methylenetetrahydrofolate reductase 677C-->T genotypes. Asia Pac J Clin Nutr. 2008; 17(2):330-8. PMID: 18586656
101. Kou MC, Wang L, Liang KJ, Wu MJ. Genotyping 5,10-Methylenetetrahydrofolate Reductase for Patients with Coronary Artery Disease in Southern Taiwan. J Food Drug Anal. 2001; 9(3):173-7.
102. Hsu LA, Ko YL, Wang SM, Chang CJ, Hsu TS, Chiang CW, et al. The C677T mutation of the methylenetetrahydrofolate reductase gene is not associated with the risk of coronary artery disease or venous thrombosis among Chinese in Taiwan. Hum Hered. 2001; 51(1-2):41-5. https://doi.org/22958 PMID: 11096270
103. Chen TY, Chen JH, Tsao CJ. Methylenetetrahydrofolate reductase gene polymorphism and coronary artery disease in Taiwan Chinese. Haematologica. 2000; 85(4):445-6. PMID: 10756383
104. Chao CL, Tsai HH, Lee CM, Hsu SM, Kao JT, Chien KL, et al. The graded effect of hyperhomocysteinemia on the severity and extent of coronary atherosclerosis. Atherosclerosis. 1999; 147(2):379-86. PMID: 10559524
105. Chen W, Hua K, Gu H, Zhang J, Wang L. Methylenetetrahydrofolate reductase C667T polymorphism is associated with increased risk of coronary artery disease in a Chinese population. Scand J Immunol. 2014; 80(5):346-53. https://doi.org/10.1111/sji.12215 PMID: 25124382
106. Yu X, Liu J, Zhu H, Xia Y, Gao L, Dong Y, et al. Synergistic association of DNA repair relevant gene polymorphisms with the risk of coronary artery disease in northeastern Han Chinese. Thromb Res. 2014; 133(2):229-34. https://doi.org/10.1016/j.thromres.2013.11.017 PMID: 24315498
107. Yang LY, He Y, Yang DZ. Detection of homocysteine metabolism related enzymes polymorphisms in Han population with coronary heart disease in Henan province. J Zhengzhou Univ Med Sci Ed. 2011; 46(1):67-70.
108. Luo D, Yan SK, Wei LZ. Relationship between polymorphism of homocysteine metabolism-related key enzyme (*MTHFR* C677T and CBS844 ins68) and type 2 diabetes mellitus patients complicating coronary heart disease. Chin J Gerontol. 2007; 27(6):541-3.
109. Chen YL, Zhang XY, Xu XJ. Correlation of the polymorphism of methylenetetrahydrofolate reductase gene and plasma homocysteine with coronary heart disease in Uygur and Han ethnic groups in Xinjiang. J Clin Rehabilitative Tissue Eng Res. 2007; 11(17):3206-9.
110. Niu J, Zhang Z, Chen MZ. Clinical study on effect of common methylenetetrahydrofolate reductase gene

mutation on coronary artery disease in hypertension. Chin J Interv Cardiol. 2005; 13(1):25-7.
111. Mu H, Chen X. Methylenetetrahydrofolate reductase gene mutation with AMI and stroke. Sect Clin Biochem Lab Med Foreign Med Sci. 2005; 26(3):145-7.
112. Sun J, Xu Y, Xue J, Zhu Y, Lu H. Methylenetetrahydrofolate reductase polymorphism associated with susceptibility to coronary heart disease in Chinese type 2 diabetic patients. Mol Cell Endocrinol. 2005; 229(1-2):95-101. https://doi.org/10.1016/j.mce.2004.09.003 PMID: 15607533
113. Jiang BQ, Zhu GM, Bao QQ. Methylenetetrahydrofolate reductase polymorphism associated with susceptibility to coronary heart disease in Chinese type 2 diabetic patients. J Shandong Univ Health Sci Ed. 2004; 42(5):619-20.
114. Fang LG, Zhu WL, Zhu GJ. Methylenetetrahydrofolate reductase gene polymorphism, homocysteine, folate and coronary artery disease. Chin J Cardiol. 2002; 30(9):515-9.
115. Mao YM, Zhao FM, Qin Q. Association of methylenetetrahydrofolate reductase gene polymorphism, level of homocysteine and coronary heart disease. Tianjin Med J. 2002; 30(8):451-3.
116. Yu J, Chen B, Zhang G, Fu S, Li P. The 677 C-->T mutation in the methylenetetrahydrofolate reductase (*MTHFR*) gene in five Chinese ethnic groups. Hum Hered. 2000; 50(4):268-70. https://doi.org/22929 PMID: 10782023
117. Xu HY, Chen ZJ, Tang J. C677T genetic polymorphism of methylenetetrahydrofolate reductase in premature coronary heart disease. Acta Acad Med Sin. 1999; 21(2):118-21.
118. Li XB, Li Y, Li YP. The relationship between coronary heart disease and methylenetetrahydrofolate reductase in elderly. China Pract Med. 2010; 5(12):24-5.
119. Li LY, Jiang DQ, Liu ZY. Concentration of plasma homocysteine and the gene types of methylenetetrahydrofolate reductase C677T in patients with coronary heart disease and their clinical significance. Chin J Arterioscler. 2005; 13(2):210-4.
120. Gao QQ, Xu LX, Zhang HQ. Relationship between folate and methylenetetrahydrofolate reductase gene C677T polymorphisms and acute myocardial infarction. J Wenzhou Med Coll. 2004; 34(6):423-5.
121. Zhang G, Dai C. Gene polymorphisms of homocysteine metabolism-related enzymes in Chinese patients with occlusive coronary artery or cerebral vascular diseases. Thromb Res. 2001; 104(3):187-95. PMID: 11672761
122. Zheng YZ, Tong J, Do XP, Pu XQ, Zhou BT. Prevalence of methylenetetrahydrofolate reductase C677T and its association with arterial and venous thrombosis in the Chinese population. Br J Haematol. 2000; 109(4):870-4. PMID: 10929044
123. Dogra RK, Das R, Ahluwalia J, Kumar RM, Talwar KK. Prothrombotic gene polymorphisms and plasma factors in young North Indian survivors of acute myocardial infarction. J Thromb Thrombolysis. 2012; 34(2):276-82. https://doi.org/10.1007/s11239-012-0734-6 PMID: 22535530
124. Gupta SK, Kotwal J, Kotwal A, Dhall A, Garg S. Role of homocysteine & *MTHFR* C677T gene polymorphism as risk factors for coronary artery disease in young Indians. Indian J Med Res. 2012; 135(4):506-12. PMID: 22664498
125. Kanth VV, Golla JP, Sastry BK, Naik S, Kabra N, Sujatha M. Genetic interactions between *MTHFR* (C677T), methionine synthase (A2756G, C2758G) variants with vitamin B12 and folic acid determine susceptibility to premature coronary artery disease in Indian population. J Cardiovasc Dis Res. 2011; 2(3):156-63. https://doi.org/10.4103/0975-3583.85262 PMID: 22022143
126. Dayakar S, Goud KI, Reddy TP, Rao SP, Sesikeran SB, Sadhnani M. Sequence variation of the methylene tetrahydrofolate reductase gene (677C>T and 1298 A>C) and traditional risk factors in a South Indian population. Genet Test Mol Biomarkers. 2011; 15(11):765-9. https://doi.org/10.1089/gtmb.2011.0024 PMID: 21749215
127. Vijaya Lakshmi SV, Naushad SM, Rupasree Y, Seshagiri Rao D, Kutala VK. Interactions of 5'-UTR thymidylate synthase polymorphism with 677C --> T methylene tetrahydrofolate reductase and 66A --> G methyltetrahydrofolate homocysteine methyl-transferase reductase polymorphisms determine susceptibility to coronary artery disease. J Atheroscler Thromb. 2011; 18(1):56-64. PMID: 20962453
128. Dhar S, Chatterjee S, Ray S, Dutta A, Sengupta B, Chakrabarti S. Polymorphisms of methylenetetrahydrofolate reductase gene as the genetic predispositions of coronary artery diseases in eastern India. J Cardiovasc Dis Res. 2010; 1(3):152-7. https://doi.org/10.4103/0975-3583.70922 PMID: 21187870
129. Angeline T, Jeyaraj N, Tsongalis GJ. *MTHFR* Gene polymorphisms, B-vitamins and hyperhomocystinemia in young and middle-aged acute myocardial infarction patients. Exp Mol Pathol. 2007; 82(3):227-33. https://doi.org/10.1016/j.yexmp.2007.02.005 PMID: 17412321
130. Mukherjee M, Joshi S, Bagadi S, Dalvi M, Rao A, Shetty KR. A low prevalence of the C677T mutation in the methylenetetrahydrofolate reductase gene in Asian Indians. Clin Genet. 2002; 61(2):155-9. PMID: 11940092
131. Iqbal MP, Fatima T, Parveen S, Yousuf FA, Shafiq M, Mehboobali N, et al. Lack of association of methylenetetrahydrofolate reductase 677C>T mutation with coronary artery disease in a Pakistani population. J

Mol Genet Med. 2005; 1(1):26-32. PMID: 19565010

132. Heidari MM, Khatami M, Hadadzadeh M, Kazemi M, Mahamed S, Malekzadeh P, et al. Polymorphisms in NOS3, *MTHFR*, APOB and TNF-alpha Genes and Risk of Coronary Atherosclerotic Lesions in Iranian Patients. Res Cardiovasc Med. 2016; 5(1):e29134. https://doi.org/10.5812/cardiovascmed.29134 PMID: 26878010
133. Abu-Amero KK, Wyngaard CA, Dzimiri N. Prevalence and role of methylenetetrahydrofolate reductase 677 C-->T and 1298 A-->C polymorphisms in coronary artery disease in Arabs. Arch Pathol Lab Med. 2003; 127(10):1349-52. https://doi.org/10.1043/1543-2165(2003)127<1349:paromr>2.0.co;2 PMID: 14521457
134. Ilhan N, Kucuksu M, Kaman D, Ilhan N, Ozbay Y. The 677 C/T *MTHFR* polymorphism is associated with essential hypertension, coronary artery disease, and higher homocysteine levels. Arch Med Res. 2008; 39(1):125-30. https://doi.org/10.1016/j.arcmed.2007.07.009 PMID: 18068006
135. Gulec S, Aras O, Akar E, Tutar E, Omurlu K, Avci F, et al. Methylenetetrahydrofolate reductase gene polymorphism and risk of premature myocardial infarction. Clin Cardiol. 2001; 24(4):281-4. PMID: 11303694
136. Tokgozoglu SL, Alikasifoglu M, Unsal, Atalar E, Aytemir K, Ozer N, et al. Methylene tetrahydrofolate reductase genotype and the risk and extent of coronary artery disease in a population with low plasma folate. Heart. 1999; 81(5):518-22. PMID: 10212171
137. Almawi WY, Ameen G, Tamim H, Finan RR, Irani-Hakime N. Factor V G1691A, prothrombin G20210A, and methylenetetrahydrofolate reductase [*MTHFR*] C677T gene polymorphism in angiographically documented coronary artery disease. J Thromb Thrombolysis. 2004; 17(3):199-205. https://doi.org/10.1023/b:thro.0000040489.86029.27 PMID: 15353918
138. Mager A, Lalezari S, Shohat T, Birnbaum Y, Adler Y, Magal N, et al. Methylenetetrahydrofolate reductase genotypes and early-onset coronary artery disease. Circulation. 1999; 100(24):2406-10. PMID: 10595952
139. El-Sammak M, Kandil M, El-Hifni S, Hosni R, Ragab M. Elevated plasma homocysteine is positively associated with age independent of C677T mutation of the methylenetetrahydrofolate reductase gene in selected Egyptian subjects. Int J Med Sci. 2004; 1(3):181-92. PMID: 15912197
140. Ghazouani L, Abboud N, Mtiraoui N, Zammiti W, Addad F, Amin H, et al. Homocysteine and methylenetetrahydrofolate reductase C677T and A1298C polymorphisms in Tunisian patients with severe coronary artery disease. J Thromb Thrombolysis. 2009; 27(2):191-7. https://doi.org/10.1007/s11239-008-0194-1 PMID: 18204887
141. Kerkeni M, Addad F, Chauffert M, Myara A, Gerhardt M, Chevenne D, et al. Hyperhomocysteinaemia, methylenetetrahydrofolate reductase polymorphism and risk of coronary artery disease. Ann Clin Biochem. 2006; 43(Pt 3):200-6. https://doi.org/10.1258/000456306776865232 PMID: 16704755
142. Bennouar N, Allami A, Azeddoug H, Bendris A, Laraqui A, El Jaffali A, et al. Thermolabile methylenetetrahydrofolate reductase C677T polymorphism and homocysteine are risk factors for coronary artery disease in Moroccan population. J Biomed Biotechnol. 2007; 2007(1):80687. https://doi.org/10.1155/2007/80687 PMID: 17497026
143. Ramkaran P, Phulukdaree A, Khan S, Moodley D, Chuturgoon AA. Methylenetetrahydrofolate reductase C677T polymorphism is associated with increased risk of coronary artery disease in young South African Indians. Gene. 2015; 571(1):28-32. https://doi.org/10.1016/j.gene.2015.06.044 PMID: 26095803
144. Ranjith N, Pegoraro RJ, Rom L. Risk factors and methylenetetrahydrofolate reductase gene polymorphisms in a young South African Indian-based population with acute myocardial infarction. Cardiovasc J S Afr.. 2003; 14(3):127-32. PMID: 12844196

Supplementary Table S2. Pooled analysis: *MTHFR* C677T genotypes and risks of coronary artery disease (CAD) by ethnicity (93 Studies).

Genotypes (Number of studies)	CAD Cases (*N* = 22,994) *n* (%)		CAD Controls (*N* = 20,221) *n* (%)		Test of Heterogeneity			Test of Association	
					Q	*p*	I^2	Risk Ratio* (95% CI)	*p*
**TT (93)**	2,959	(12.87)	2,043	(10.10)	188.95	<0 .0001	51.3%	1.27 [1.16, 1.38]	<0.0001
Caucasian (47)	1,574	(11.39)	1,071	(10.01)	43.94	0.5588	0%	1.10 [1.02, 1.18]	0.0166
East Asian (25)	1,012	(20.84)	746	(15.13)	50.15	0.0014	52.1%	1.30 [1.12, 1.50]	0.0004
South Asian (8)	73	(5.04)	51	(3.15)	13.44	0.062	47.9%	1.67 [1.20, 2.32]	0.0024
Mixed (2)	86	(9.75)	45	(8.75)	0.00	0.9658	0%	0.87 [0.62, 1.23]	0.43
Mid-Eastern (6)	115	(9.84)	84	(5.18)	27.05	< 0.0001	81.5%	2.56 [1.22, 5.36]	0.013
African (5)	99	(12.01)	46	(5.54)	1.51	0.8251	0%	2.22 [1.59, 3.10]	<0.0001
**CT (93)**	9,972	(43.37)	8,242	(40.76)	130.76	0.0049	29.6%	1.04 [1.01, 1.07]	0.0074
Caucasian (47)	6,177	(44.71)	4,671	(43.65)	42.77	0.6084	0%	1.01 [0.98, 1.04]	0.61
East Asian (25)	2,265	(46.65)	2,248	(45.59)	40.13	0.0207	40.2%	1.04 [0.98, 1.11]	0.19
South Asian (8)	387	(26.74)	301	(18.57)	6.69	0.4614	0%	1.36 [1.19, 1.55]	<0.0001
Mixed (2)	381	(43.20)	186	(36.19)	0.02	0.8918	0%	0.99 [0.86, 1.12]	0.83
Mid-Eastern (6)	450	(38.49)	558	(34.38)	5.67	0.3392	11.9%	1.18 [1.06, 1.31]	0.0016
African (5)	312	(37.86)	278	(33.49)	9.15	0.0574	56.3%	1.14 [1.00, 1.30]	0.053
**CC (93)**	10,063	(43.76)	9,936	(49.14)	198.99	< 0.0001	53.8%	0.91 [0.88, 0.94]	<0.0001
Caucasian (47)	6,066	(43.90)	4,960	(46.35)	50.56	0.2981	9%	0.97 [0.94, 1.00]	0.0458
East Asian (25)	1,578	(32.50)	1,937	(39.28)	63.69	< 0.0001	62.3%	0.82 [0.75, 0.91]	< .0001
South Asian (8)	987	(68.21)	1,269	(78.29)	20.79	0.0041	66.3%	0.89 [0.83, 0.95]	0.0008
Mixed (2)	415	(47.05)	283	(55.06)	0.54	0.4626	0%	1.04 [0.93, 1.15]	0.49
Mid-Eastern (6)	604	(51.67)	981	(60.44)	15.34	0.009	67.4%	0.76 [0.65, 0.88]	0.0004
African (5)	413	(50.12)	506	(60.96)	11.80	0.0189	66.1%	0.87 [0.74, 1.02]	0.086
**TT+CT (93)**	12,931	(56.24)	10,285	(50.86)	214.72	<0.0001	57.2%	1.09 [1.06, 1.13]	<0.0001
**CC+CT (93)**	20,035	(87.13)	18,178	(89.90)	230.18	<0.0001	60%	0.98 [0.97, 0.99]	<0.0001
**T (93)**	7,945	(34.55)	6,164	(30.48)	137.27	0.0016	33%	1.12 [1.08, 1.16]	<0.0001
**C (93)**	15,049	(65.45)	14,057	(69.52)	127.50	0.0085	27.8%	0.95 [0.94, 0.97]	<0.0001

Note. Q = Cochran's Q; CI = confidence interval; *The fixed-effect model was used if $p > .05$, the random-effects model was used if $p < .05$ based on the test of heterogeneity.

Supplementary Table S3. Pooled analysis: *MTHFR* C677T genotypes and risks of myocardial infarction (MI) by ethnicity (30 Studies).

Genotype by Race or Ethnicity (Number of studies)	MI Cases (*N* = 6,709) *n* (%)	MI Controls (*N* = 10,807) *n* (%)	Test of Heterogeneity			Test of Association	
			Q	*p*	I^2	Risk Ratio* (95% CI)	*p*
TT (30)	853 (12.73)	1,287 (11.91)	47.01	0.0186	38.3%	1.13 [0.99, 1.28]	0.079
Caucasian (13)	276 (9.68)	408 (9.66)	13.61	0.3262	11.8%	0.97 [0.84, 1.12]	0.68
East Asian (7)	427 (18.92)	697 (15.35)	10.53	0.1041	43%	1.30 [1.14, 1.46]	<0.0001
South Asian (5)	20 (1.95)	20 (1.75)	5.52	0.2381	27.5%	1.12 [0.63, 2.01]	0.70
Mixed (1)	7 (10.14)	43 (12.72)	--	--	--	0.80	--
Mid-Eastern (1)	15 (15.63)	5 (5.00)	--	--	--	3.13	--
Hispanic (2)	102 (28.90)	106 (29.12)	1.50	0.2204	33.4%	0.99 [0.79, 1.25]	0.94
African (1)	6 (12.00)	8 (8.00)	--	--	--	1.50	--
CT (30)	2,805 (41.85)	4,573 (42.32)	43.19	0.0438	32.8%	1.04 [0.98, 1.09]	0.1831
Caucasian (13)	1,225 (42.98)	1,777 (42.08)	4.81	0.9641	0%	1.02 [0.96, 1.07]	0.58
East Asian (7)	1,073 (47.54)	2,191 (48.26)	12.61	0.0496	52.4%	1.00 [0.90, 1.12]	0.997
South Asian (5)	247 (24.03)	230 (20.14)	18.56	0.001	78.5%	1.27 [0.86, 1.88]	0.23
Mixed (1)	34 (49.28)	141 (41.72)	--	--	--	1.18	--
Mid-Eastern (1)	39 (40.63)	35 (35.00)	--	--	--	1.16 [0.81, 1.66]	--
Hispanic (2)	165 (46.74)	157 (43.13)	1.88	0.1709	46.7%	1.08 [0.92, 1.27]	0.33
African (1)	22 (44.00)	42 (42.00)	--	--	--	1.05	--
CC (30)	3,045 (45.43)	4,947 (45.78)	67.52	<0.0001	57%	0.93 [0.89, 0.99]	0.0148
Caucasian (13)	1,349 (47.33)	2,038 (48.26)	12.41	0.4135	3.3%	0.99 [0.94, 1.04]	0.76
East Asian (7)	757 (33.54)	1,652 (36.39)	24.59	0.0004	75.6%	0.86 [0.72, 1.03]	0.098
South Asian (5)	761 (74.03)	892 (78.11)	22.43	0.0002	82.2%	0.94 [0.84, 1.05]	0.29
Mixed (1)	28 (40.58)	154 (45.56)	--	--	--	0.89	--
Mid-Eastern (1)	42 (43.75)	60 (60.00)	--	--	--	0.73	--
Hispanic (2)	86 (24.36)	101 (27.75)	0.04	0.8487	0%	0.88 [0.69, 1.13]	0.31
African (1)	22 (44.00)	50 (50.00)	--	--	--	0.88	--
TT+CT (30)	3,658 (54.57)	5,860 (54.22)	64.84	0.0001	55.3%	1.06 [1.01, 1.12]	0.0187
CC+CT (30)	5,850 (87.27)	9,520 (88.09)	67.56	<0.0001	57.1%	0.99 [0.97, 1.00]	0.063
T (30)	2,224 (33.70)	3,567 (33.10)	40.64	0.074	29.6%	1.06 [1.02, 1.11]	0.0073
C (30)	4,376 (66.30)	7,211 (66.90)	42.99	0.0456	32.5%	0.97 [0.94, 1.00]	0.0223

Note. Q = Cochran's Q; CI = confidence interval; *The fixed-effect model was used if $p > .05$, the random-effects model was used if $p < .05$ based on the test of heterogeneity.

(a)

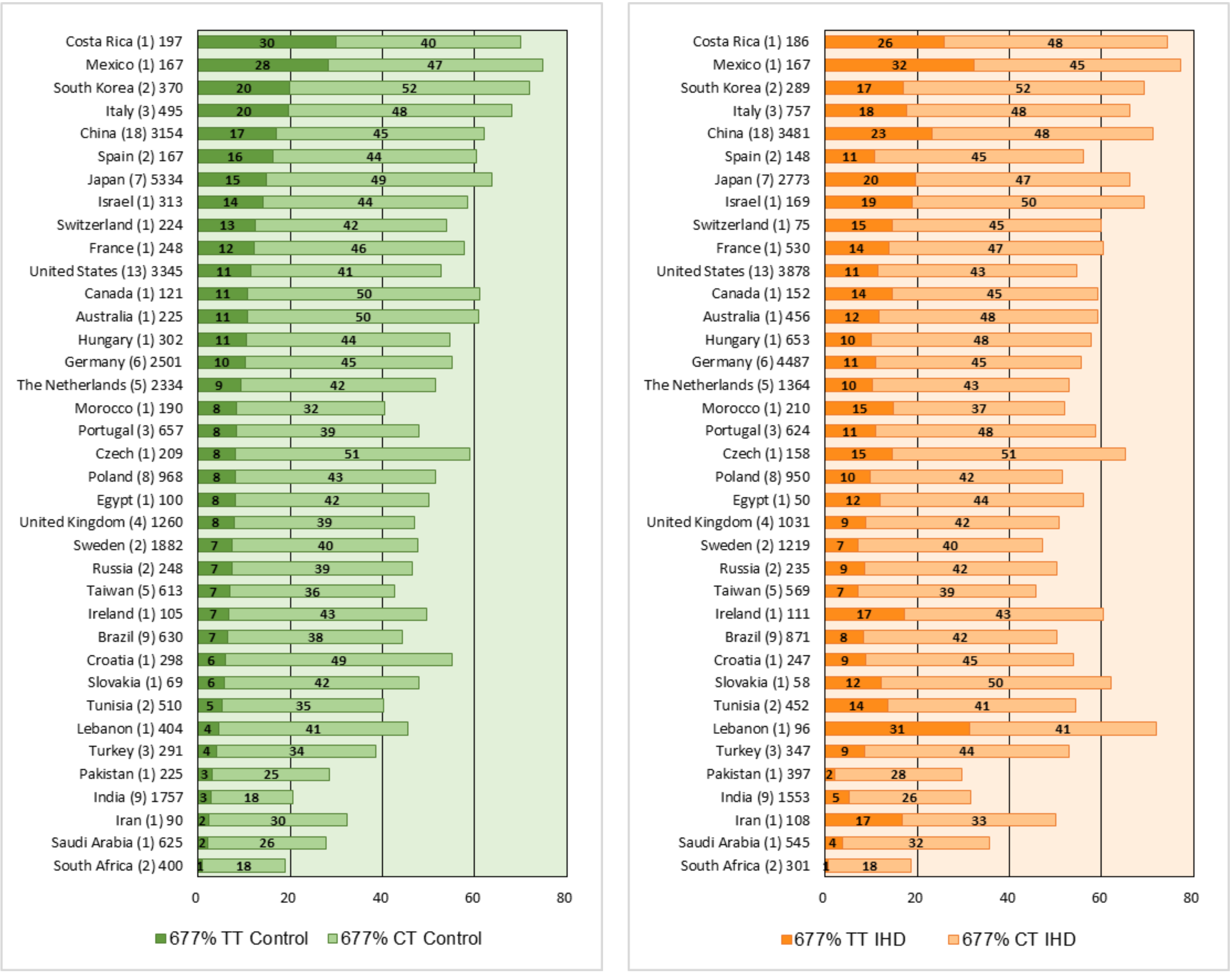

(b)

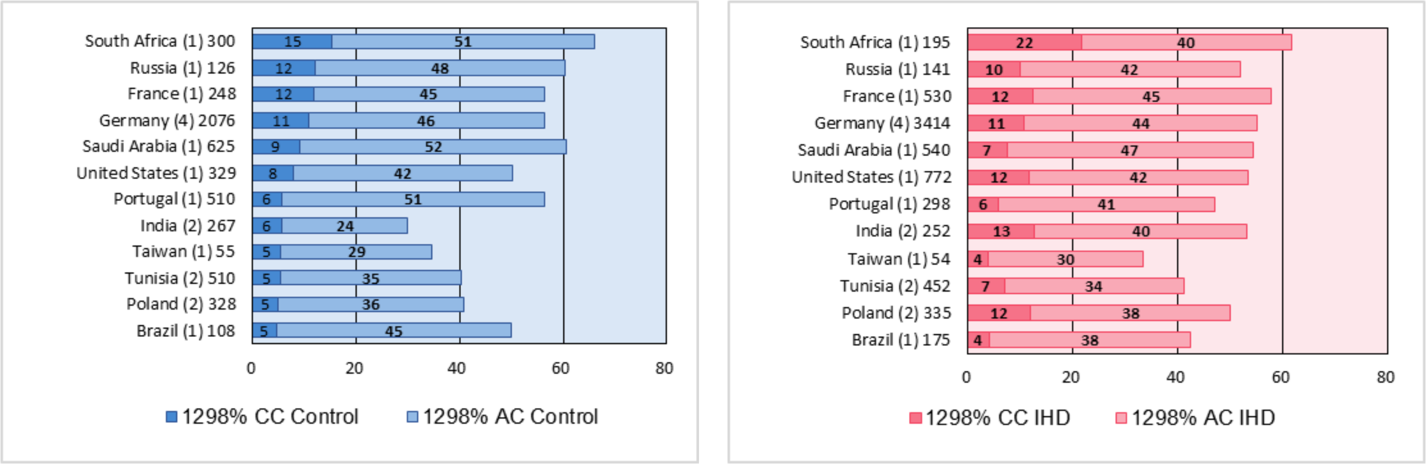

Supplementary Figure S1a. *MTHFR* C677T percentage of polymorphism per control and IHD case groups; **S1b.** *MTHFR* A1298C percentage of polymorphism per control and IHD case groups.

(a)

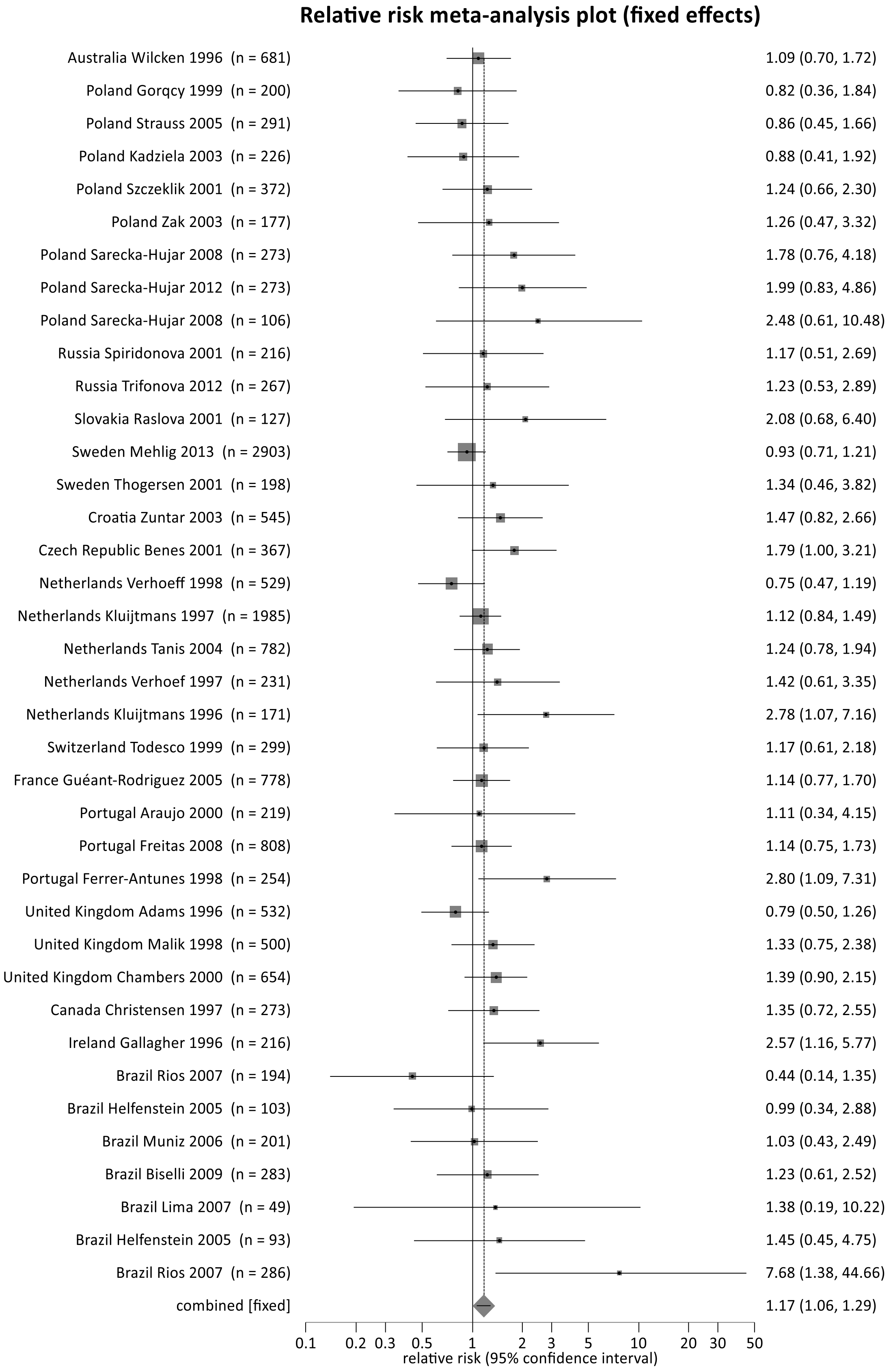

Supplementary Figure S2a. Forest plot for meta-analysis of *MTHFR* 677 polymorphism by TT genotype, countries of Caucasian with risks >1.

(b)

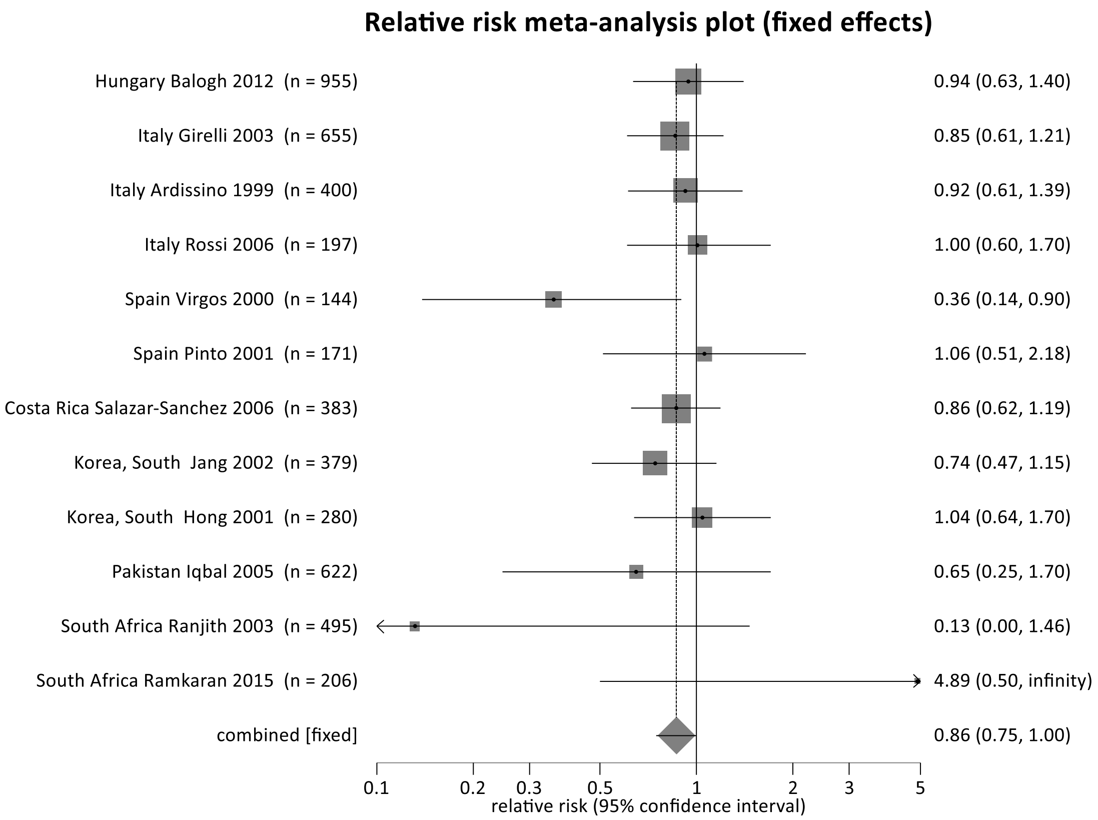

Supplementary Figure S2b. Forest plot for meta-analysis of *MTHFR* 677 polymorphism by TT genotype, countries with risks < 1.

(c)

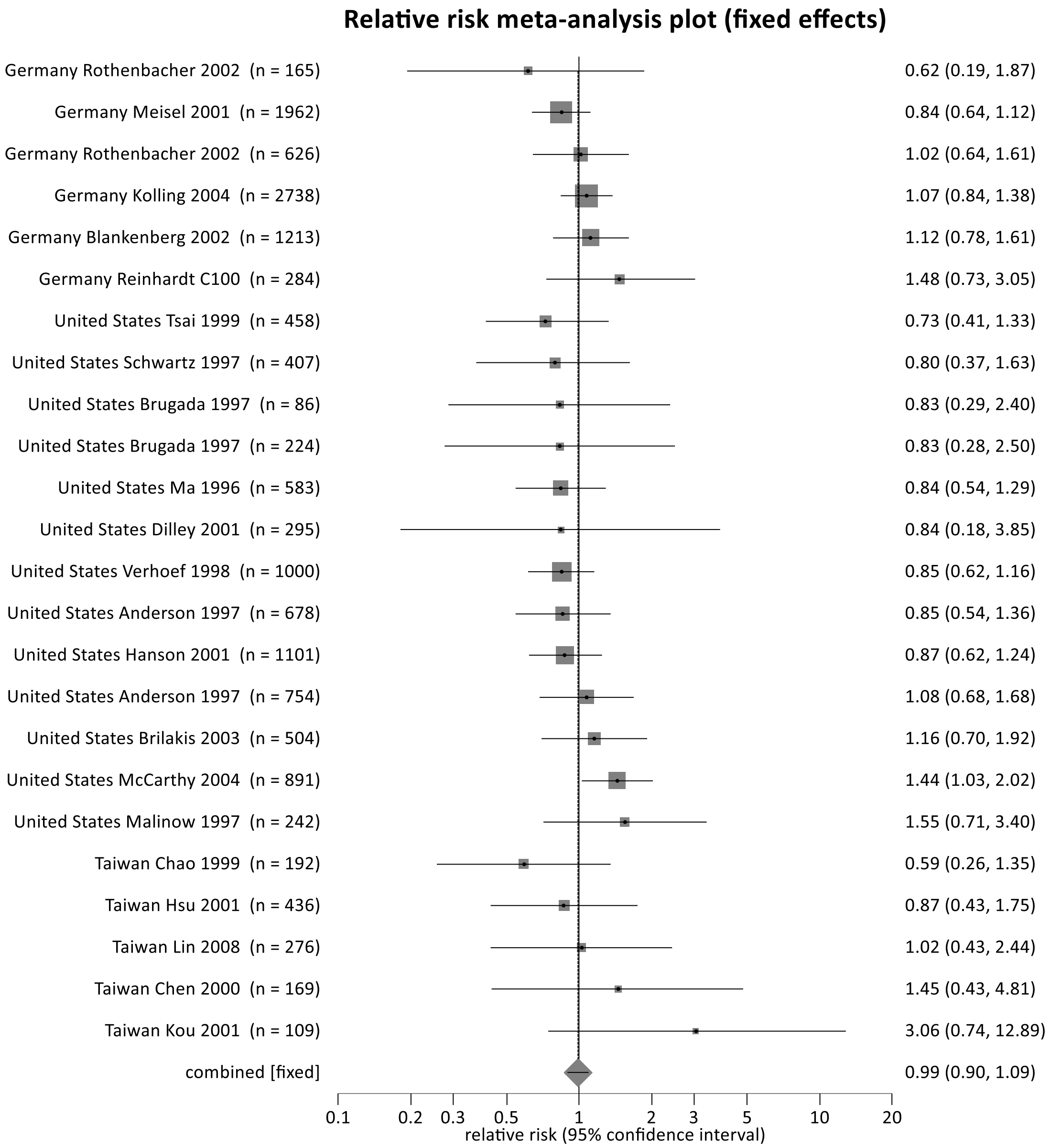

Supplementary Figure S2c. Forest plot for meta-analysis of *MTHFR* 677 polymorphism by TT genotype, countries with risks varied around 1.

(a)

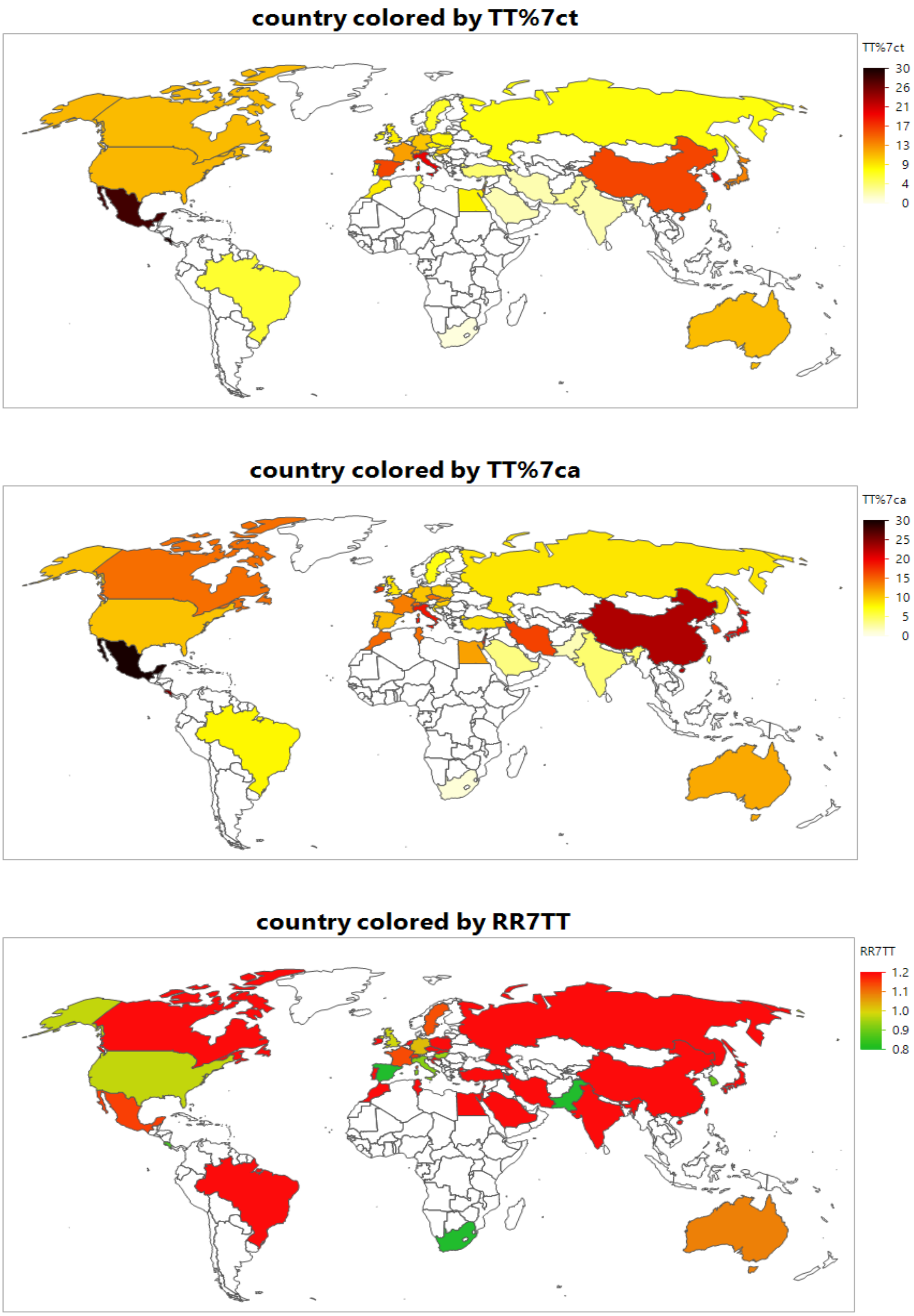

Supplementary Figure S3a. Geographic information maps for percentages of *MTHFR* 677 TT genotype per control (top) and IHD case groups (middle), and its association with IHD risks (bottom).

Note. TT%7ct: percentage of *MTHFR* 677 TT genotype in control group; TT%7ca: percentage of *MTHFR* 677 genotype in case group; RR7TT: the relative risk between percentage of *MTHFR* 677 genotypes per case/control. TTCT%7ct: percentage of *MTHFR* 677 TT plus CT genotypes in control group; TTCT%7ca: percentage of *MTHFR* 677 TT plus CT genotypes in case group; RR7TTCT: the relative risk between percentage of *MTHFR* 677 TT plus CT genotypes and development of IHD.

(b)

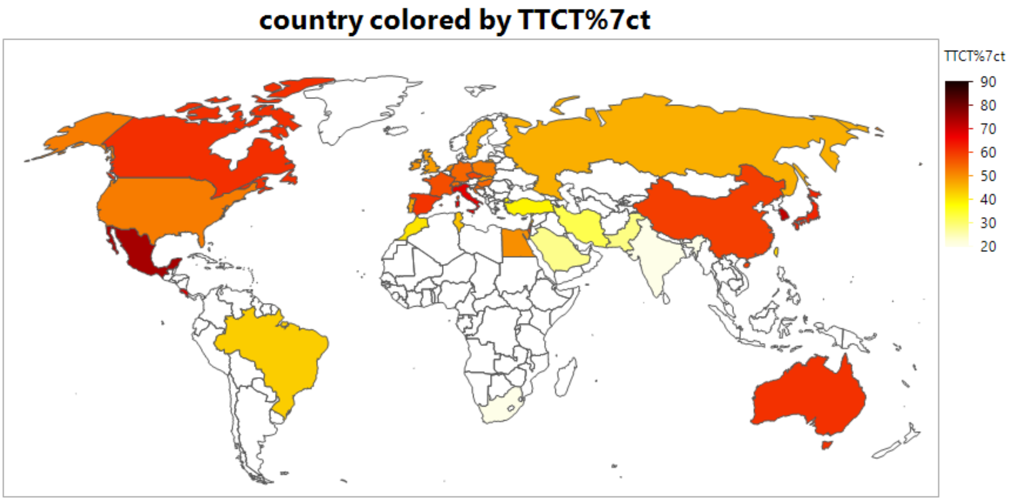

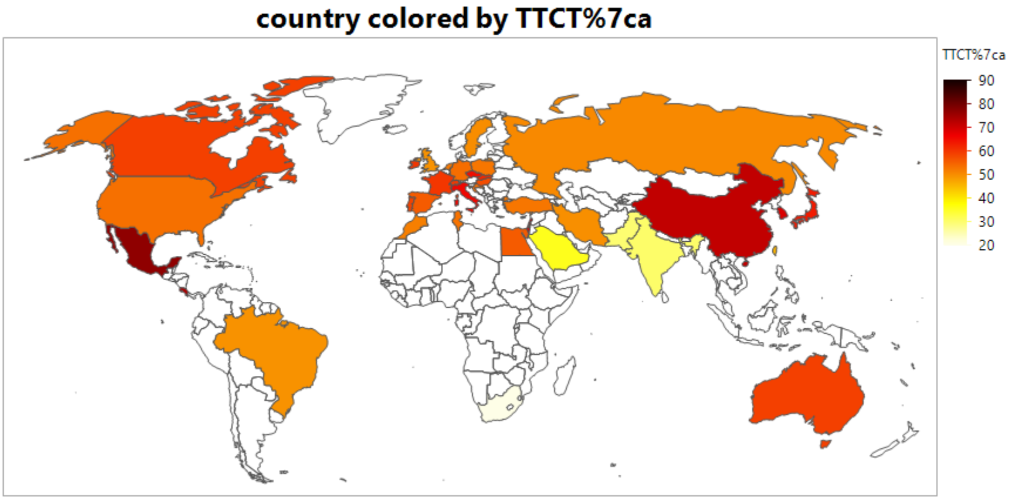

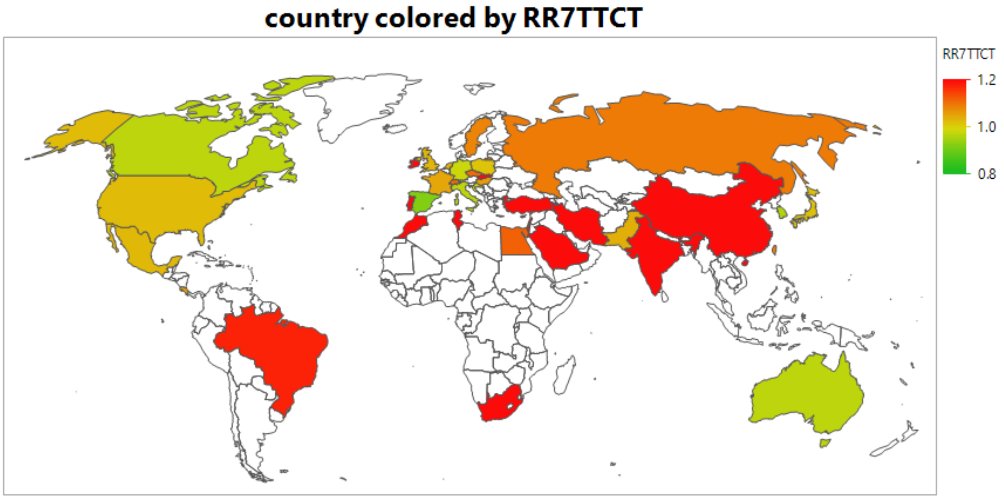

Supplementary Figure S3b. Geographic information maps for percentages of *MTHFR* 677 TT plus CT genotypes per control (top) and ischemic heart disease (IHD) case groups (middle), and their associations with IHD risks (bottom). Note. TT%7ct: percentage of *MTHFR* 677 TT genotype in control group; TT%7ca: percentage of *MTHFR* 677 genotype in case group; RR7TT: the relative risk between percentage of *MTHFR* 677 genotypes per case/control. TTCT%7ct: percentage of *MTHFR* 677 TT plus CT genotypes in control group; TTCT%7ca: percentage of *MTHFR* 677 TT plus CT genotypes in case group; RR7TTCT: the relative risk between percentage of *MTHFR* 677 TT plus CT genotypes and development of IHD.
